# A systems pharmacology-oriented discovery of a new therapeutic use of the TCM formula Liuweiwuling for liver failure

**DOI:** 10.1038/s41598-018-21515-6

**Published:** 2018-04-04

**Authors:** Jia-bo Wang, He-rong Cui, Rui-lin Wang, Cong-en Zhang, Ming Niu, Zhao-fang Bai, Gen-hua Xu, Peng-yan Li, Wen-yan Jiang, Jing-jing Han, Xiao Ma, Guang-ming Cai, Rui-sheng Li, Li-ping Zhang, Xiao-he Xiao

**Affiliations:** 1 China Military Institute of Chinese Medicine, 302 Military Hospital, Beijing, PR China; 2 Traditional Chinese Medicine College, Beijing University of Traditional Chinese Medicine, Beijing, PR China; 3 Integrative Medicine Center, 302 Military Hospital, Beijing, PR China; 4 College of Pharmacy, Chengdu University of Traditional Chinese Medicine, Chengdu, PR China; 5 Research Center for Clinical and Translational Medicine, 302 Hospital of PLA, Beijing, PR China

## Abstract

Multiple components of traditional Chinese medicine (TCM) formulae determine their treatment targets for multiple diseases as opposed to a particular disease. However, discovering the unexplored therapeutic potential of a TCM formula remains challenging and costly. Inspired by the drug repositioning methodology, we propose an integrated strategy to feasibly identify new therapeutic uses for a formula composed of six herbs, Liuweiwuling. First, we developed a comprehensive systems approach to enrich drug compound-liver disease networks to analyse the major predicted diseases of Liuweiwuling and discover its potential effect on liver failure. The underlying mechanisms were subsequently predicted to mainly attribute to a blockade of hepatocyte apoptosis via a synergistic combination of multiple effects. Next, a classical pharmacology experiment was designed to validate the effects of Liuweiwuling on different models of fulminant liver failure induced by D-galactosamine/lipopolysaccharide (GalN/LPS) or thioacetamide (TAA). The results indicated that pretreatment with Liuweiwuling restored liver function and reduced lethality induced by GalN/LPS or TAA in a dose-dependent manner, which was partially attributable to the abrogation of hepatocyte apoptosis by multiple synergistic effects. In summary, the integrated strategy discussed in this paper may provide a new approach for the more efficient discovery of new therapeutic uses for TCM formulae.

## Introduction

Traditional Chinese medicine (TCM) is a continuously evolving system originating from ancient medical practice that still plays an important role in the health of Asian people^1^. TCM differs from modern medicine in terms of substance, methodology and philosophy, and its ultimate goal is to cure the underlying biological pathogenesis through the functional analysis of an individual entity based on a syndrome (*Zheng* in Chinese, which includes multiple known and unknown diseases). Thus, it can be said that the treatment target of TCM is *Zheng* rather than a particular modern disease, and that is why this treatment regimen has a satisfactory effect on a variety of complex diseases, especially on paroxysmal, non-specific diseases^2–4^. Despite its growing popularity in Western countries, the main constraint on TCM promotion is defining which modern diseases (instead of *Zheng*) these traditional medicines could work in and why they work^1,5^.

The development of computational analyses and systems modelling approaches has advanced the field of systems biology^6–8^. It should be noted that^9,10^ “if there is any technology that could lead to a breakthrough in traditional Chinese medicine, it will be systems biology”. Recently, systems biology has been widely used to evaluate the interactions of proteins and small molecules in biological systems to investigate the mechanisms of TCM/formulae^11–13^, which provides a new and powerful approach for these multi-target drugs. In a previous study^14^, we constructed for the first time a “compound–target–disease” network by combining multiple drug-target predictions and disease–specific target proteins with protein-protein interactions to screen out the underlying targets and mechanisms of San-Cao granules in liver fibrosis. A network analysis of the noteworthy features could provide important information to explain the functions of certain drugs in specific diseases. Furthermore, drug repositioning predicted by the calculations based on systems biology has recently become a research focus in computational biology and systems biology^15–17^. The strategy of integrating classical pharmacology with systems biology has the potential to provide a better strategy for identifying new therapeutic uses and mechanisms for TCM formulae.

**Figure 1:**
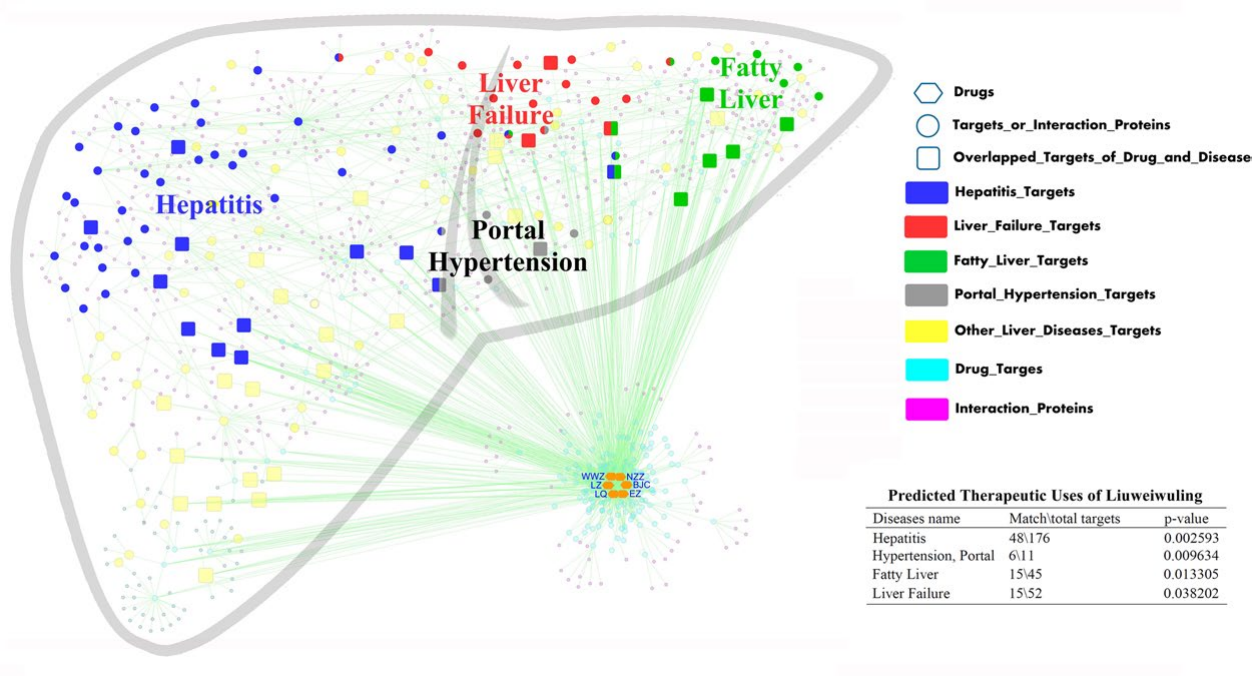
Discovery of new therapeutic use of Liuweiwuling by drug repositioning in liver disease by network pharmacology. This network of compound–target–disease was enriched to analyse the major predicted diseases of Liuweiwuling by using the hypergeometric p-value with a condition of p-value < 0.05 and matching count> 2. Notes: The hexagon nodes represent the six drugs of Liuweiwuling, the circle nodes represent the human proteins (including drug targets, disease targets and interaction proteins) and the square nodes represent the common protein targets of herbs and liver diseases. To discriminate the proteins, drug targets were colored in azure, hepatitis related targets were colored in blue, liver failure related targets were colored in red, fatty liver related targets were colored in green, portal hypertension related targets were colored in gray, other diseases related targets were colored in yellow and the interaction proteins were colored in purple. For the abbreviation of six drugs in Liuweiwuling, Schisandrae Chinensis Fructus (WWZ), Ligustri Lucidi Fructus (NZZ), Forsythiae Fructus (LQ), Sonchus brachyotus DC. (BJC), Curcumae Rhizoma (EZ) and Ganoderma Spore (LZ).

This purpose of this study was to propose a strategy for the discovery of new therapeutic uses for TCM formulae by combining classical pharmacology with a comprehensive systems analysis involving the enrichment of drug compound-disease networks (see Fig. 1). In this study, we chose the classical TCM formulae Liuweiwuling to test this strategy because of its good clinical efficacy for the treatment of chronic liver injury in China^18–21^. Liuweiwuling is composed of six herbs, *Schisandrae Chinensis* Fructus (WWZ), *Ligustri Lucidi* Fructus (NZZ), *Forsythiae* Fructus (LQ), *Sonchus brachyotus* DC (BJC), *Curcumae* Rhizoma (EZ) and *Ganoderma* Spore (LZ), and formulae principal provided theoretical evidence for the effect of Liuweiwuling on liver diseases (see Supplementary Table S1). First, we developed a comprehensive systems approach to enrich drug compound-liver disease networks to analyse major predicted diseases of Liuweiwuling and to discover its potential effect on liver failure. Subsequently, the underlying mechanisms were predicted by constructing disease-specific and drug-specific networks to investigate the relationships of the putative targets and relative signal pathways between Liuweiwuling and liver failure. Additionally, formulae principal and a retrospective analysis based on data from a small sample provided theoretical and clinical evidence for the results above, indicating the preventive effect of Liuweiwuling on liver failure (see Supplementary Table S1-S4). Furthermore, the results were validated by comprehensive classical pharmacology experiments, including different models of fulminant liver failure induced by D-galactosamine/lipopolysaccharide (GalN/LPS) or thioacetamide (TAA). In summary, this study proposes an integrated strategy based on systems biology to investigate the non-specific effects of Liuweiwuling on liver failure.

## Results

### Quality control of Liuweiwuling

#### Identification of the components of Liuweiwuling

First, the chemical information of Liuweiwuling was established by UHPLC-MS in the ESI+ and ESI- modes, and the 6 components of Liuweiwuling were preliminarily identified. The UHPLC-MS identification of the components in Liuweiwuling is shown in Supplementary Fig. S1 and Table S5 in the supplementary material.

#### Multicomponent quantification of Liuweiwuling

According to the results of the above identification, the content of salidroside, phillyrin, schisandrol A, schizandrol A, schizandrin A and schizandrin B was determined by HPLC according to the described chromatographic conditions. As a result, the average concentration of salidroside, phillyrin, schisandrol A, schizandrol A, schizandrin A and schizandrin B in 4 batches of Liuweiwuling was 0.23%, 0.14%, 0.40%, 0.25%, 0.34% and 0.13%, respectively, which was in accordance with the quality standard (YBZ06972006-2009Z, the content of schizandrin A is no less than 1.0 mg/500 mg) of the Chinese National Drug Standards (2010). And few differences in 4 batches of Liuweiwuling (6 samples per batch) as shown in Supplementary Fig. S2 and Table S6, which preliminarily ensured that the sample quality was consistent.

#### Fingerprint analysis of Liuweiwuling

To further ensure the quality of Liuweiwuling, its chemical fingerprint was established. Supplementary Table S7 shows the preliminary precision and stability of this method. Four batches of Liuweiwuling were used for the fingerprint analysis, and three samples per batch were selected randomly and injected into the HPLC system. The analysis process and results are depicted in Supplementary Fig. S3 and Table S8 in the supplementary information. The results showed that the similarity values of all 12 samples were higher than 0.979, ensuring the reliability of the subsequent research.

### New therapeutic use discovery and mechanism prediction of Liuweiwuling on liver failure by network pharmacology

#### Discovery of new therapeutic use of Liuweiwuling by drug repositioning in liver disease

Based on a database construction method, a network that included a total of 219 compounds and 229 targets for Liuweiwuling and 151 predicted disease targets associated with liver disease obtained from 749 liver disease targets was established (see Fig. 1) to screen the basic diseases that Liuweiwuling might act on. Then, the hypergeometric p-value was used to indicate the major predicted diseases that Liuweiwuling might act on by referring to some Gene Ontology (GO) enrichment analysis methods. Two parameters, the p-value and the matching count, were chosen to identify the major predicted diseases. As a result, we determined the predicted diseases with a p-value < 0.05 and matching count > 2 as the major predicted diseases that Liuweiwuling might act on. As a result, (acute) liver failure, hepatitis (B), hypertension and fatty liver were identified as the major predicted diseases that Liuweiwuling might act on (see Table 1). Of these four predicted diseases, hepatitis (B) and fatty liver were known indications of this formula, then we identified liver failure as the primary disease because of its significant p-value (see Fig. 1 and Table 1). Furthermore, formulae principal and a retrospective analysis based on data from a small sample indicated that this prediction was credible (see Supplementary Tables S1–S4).

**Table 1:** Predicted therapeutic uses of Liuweiwuling by enriching compound-disease networks.

Diseases name	Match\total targets	p-value
Hepatitis	48\176	0.002593
Liver Failure, Acute	4\5	0.006421
Hypertension, Portal	6\11	0.009634
Hepatitis B	18\57	0.01261
Fatty Liver	15\45	0.013305
Liver Failure	15\52	0.038202
Hepatolenticular Degeneration	6\16	0.055747
Carcinoma, Hepatocellular	77\370	0.065914
Liver Cirrhosis	4\34	0.087592
Hepatomegaly	7\49	0.089992
Zellweger Syndrome	3\28	0.094762
Hepatitis, Autoimmune	3\7	0.116306
Liver Cirrhosis, Biliary	5\16	0.122463
Hepatitis C	13\63	0.128386
Liver Neoplasms	12\56	0.130384
Budd-Chiari Syndrome	2\4	0.1554
Hepatitis, Chronic	6\28	0.18208
Liver Cirrhosis, Alcoholic	1\1	0.201602
Peliosis Hepatis	1\1	0.201602
Hepatic Encephalopathy	2\5	0.207201
Hepatitis B, Chronic	2\6	0.248557
Hepatitis C, Chronic	1\9	0.299934
Alagille Syndrome	2\9	0.304501
Protoporphyria, Erythropoietic	1\2	0.322348
Esophageal and Gastric Varices	1\2	0.322348
Porphyrias, Hepatic	1\2	0.322348
Non-alcoholic Fatty Liver Disease	1\8	0.334275
Fatty Liver, Alcoholic	1\8	0.334275
Rift Valley Fever	1\3	0.386429
Hepatic Insufficiency	1\3	0.386429
Porphyria Cutanea Tarda	1\3	0.386429
Porphyria, Variegate	1\4	0.411638
Hepatitis A	1\4	0.411638
Reye Syndrome	1\4	0.411638

#### Identification of candidate protein targets for Liuweiwuling acting on liver failure

Based on the database construction method, a total of 219 compounds and 229 targets for Liuweiwuling and 40 candidate protein targets associated with liver failure therapy were screened (see Supplementary Fig. S4a). Among these compounds and targets for Liuweiwuling, 43 compounds and 148 targets were collected for WWZ, 30 compounds and 164 targets for NZZ, 47 compounds and 175 targets for LQ, 19 compounds and 167 targets for BJC, 33 compounds and 135 targets for EZ and 79 compounds and 157 targets for LZ. The “drug–target–disease” network was constructed using these compounds and targets along with protein-protein interactions. Three topological parameters, “Degree”, “Betweenness centrality” and “Closeness centrality” were chosen to identify the candidate liver failure targets. A hub protein node was designated if its degree was more than two times the median value of all of the nodes in the network. Then, “Betweenness centrality” and “Closeness centrality” of the hub proteins with values greater than the median value were calculated to identify candidate liver failure targets. We designated proteins with “Degree” >8, “Betweenness centrality” >0.0004 and “Closeness centrality” >0.3681 as candidate targets for liver failure therapy. As a result, ESR, LCK, HS90A, RARA, FGFR1, SRC, RXRA, HEMH, ERK2, caspase-3, MEK1 and NR1H4 were identified as the primary candidate drug targets (see Supplementary Fig. S4b and Table S9). These targets are validated by qPCR and western blotting, and most targets could be validated as indicated by differences between groups (see Supplementary Figures S5,S6).

#### Prediction of the underlying mechanisms of Liuweiwuling for liver failure

Furthermore, to explain the underlyin*g* mechanisms of Liuweiwuling on liver failure, we constructed a network composed of herb-chemical components, candidate drug targets and major liver failure targets. As shown in Supplementary Fig. S4c, the network consisted of 6 herbs, 12 candidate drug targets and 7 major liver failure targets. The major liver failure targets were used to enrich 5 KEGG pathways using the DAVID V6.8 pathway-enrichment analysis^11,12^. Considering the p-value and count, the KEGG pathway analysis indicated that apoptosis played an important role in Liuweiwuling activity on liver failure (see Table 2). It had not escaped our notice that caspase-3 (P42574) appeared in the previously mentioned list of candidate drug targets (see Supplementary Tables S9), again indicating that the preventive effect of Liuweiwuling was mainly attributable to its direct blockade of hepatocyte apoptosis. Additionally, we chose the candidate drug targets not less than the median numbers of the components in the drug connected to disease targets (the median numbers of the components in the drug connected to disease targets, respectively: 11.5 for WWZ, 2 for NZZ, 4 for LQ, 5 for BJC, 9 for EZ and 18 for LZ) to predict the primary efficacy of each composed herb. Interestingly, these major putative targets associated with different efficacies of the respective individual herbs directly or indirectly affected different targets in apoptosis, suggesting a multiple synergistic function in the action of this Chinese patent medicine on apoptosis in liver failure (see Supplementary Fig. S4d). The entire list of major liver failure targets for each individual herb in Liuweiwuling is shown in Supplementary Tables S10.

**Table 2:** Top potential pathways of Liuweiwuling on liver failure by enriching major disease targets.

Pathways	Count	p-value
Apoptosis	3	0.0012
Chagas disease (American trypanosomiasis)	3	0.0033
TNF signaling pathway	3	0.0033
Porphyrin and chlorophyll metabolism	2	0.036
p53 signaling pathway	2	0.056

### Efficacy evaluation on mortality and hepatic lesions

#### Liuweiwuling prevents GalN/LPS-induced fulminant hepatitis

**Figure 2:**
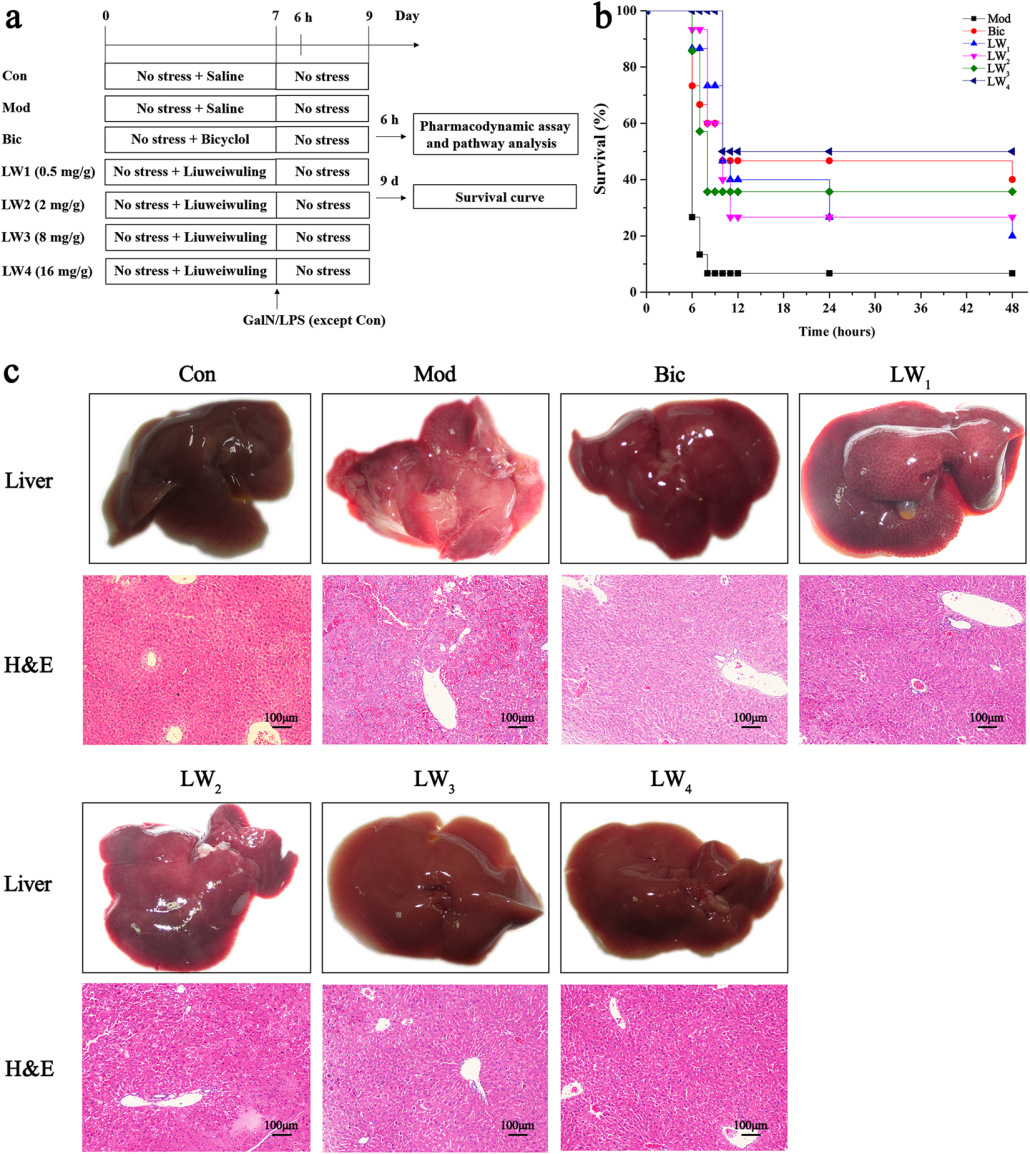
Liuweiwuling protects against GalN/LPS-induced ALF. (**a**) Experimental design for the current study. (**b**) The survival curves of the different groups (n = 15–20/group) are shown following GalN/LPS injection. (**c**) Macroscopic appearance of representative liver samples and H&E staining 6 h after GalN/LPS treatment of the different groups (n = 6–8/group) as indicated. For the abbreviation of groups in experimental design, control group (Con), model group (Mod), positive drug group (Bic), 0.5 mg/g Liuweiwuling group (LW1), 2 mg/g Liuweiwuling group (LW2), 8 mg/g Liuweiwuling group (LW3), and 16 mg/g Liuweiwuling group (LW4).

The experimental design is shown in Fig. 2a. GalN/LPS resulted in the death of PBS-pretreated mice within 48 h (see Fig. 2b) associated with hepatocellular damage, as indicated by an increase of serum aminotransferases and macroscopic and haematoxylin and eosin (H&E) histologic pathological changes in liver appearance (see Fig. 2c and Supplementary Fig. S7a). In contrast to the PBS-pretreated group, Liuweiwuling-pretreated mice survived a lethal dose of GalN/LPS in a dose-dependent manner, with fewer signs of liver injury (see Fig. 2b,c and Supplementary Fig. S7a).

#### Liuweiwuling prevents TAA-induced fulminant hepatitis

**Figure 3:**
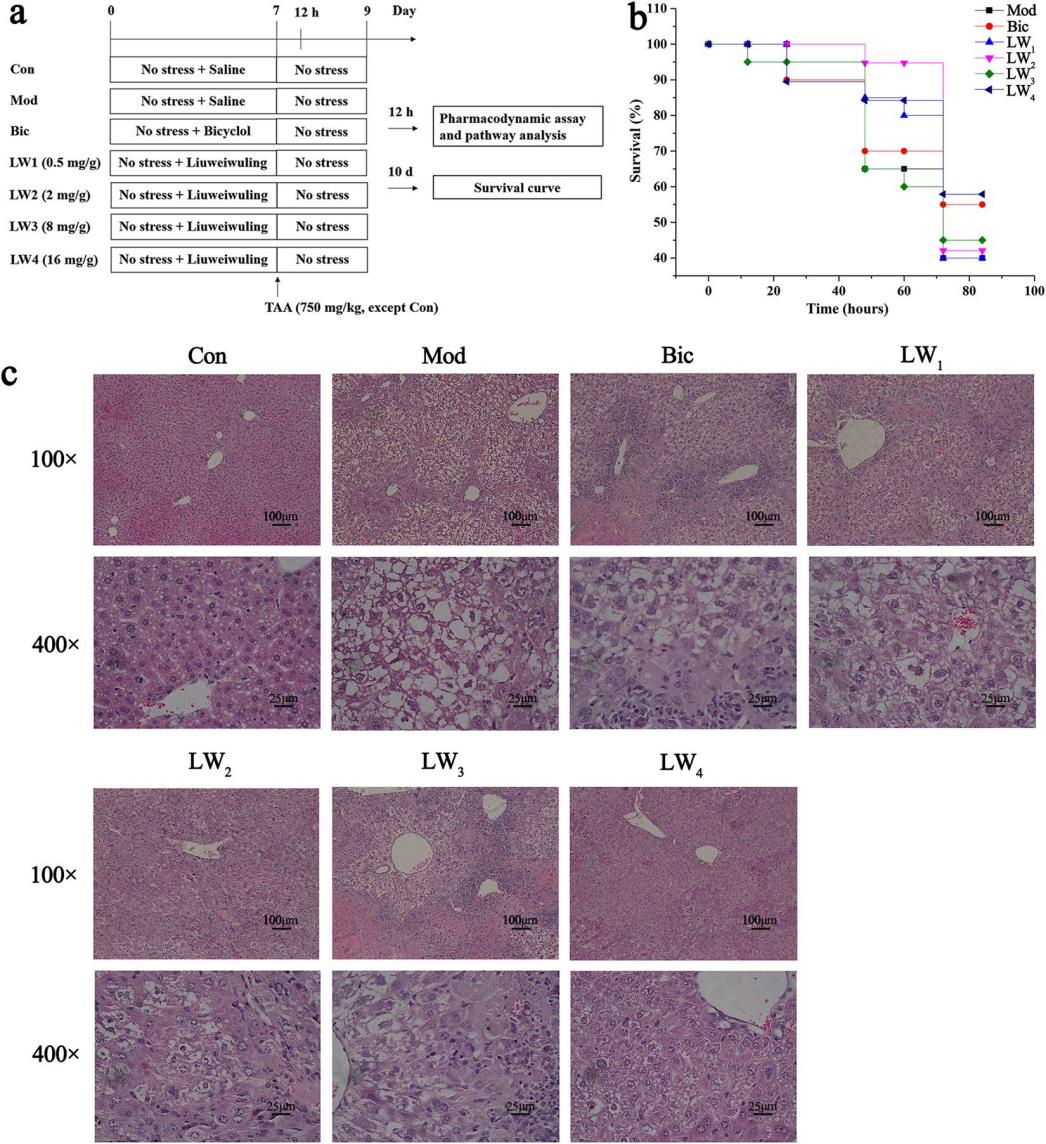
Liuweiwuling protects against TAA-induced ALF. (**a**) Experimental design for the present study. (**b**) The survival curves of the different groups (n = 15–20/group) are shown following TAA injection. (**c**) H&E staining 12 h after the last treatment with TAA for the different groups (n = 6–8/group) as indicated. For the abbreviation of groups in experimental design, control group (Con), model group (Mod), positive drug group (Bic), 0.5 mg/g Liuweiwuling group (LW1), 2 mg/g Liuweiwuling group (LW2), 8 mg/g Liuweiwuling group (LW3), and 16 mg/g Liuweiwuling group (LW4).

The experimental design is shown in Fig. 3a. TAA resulted in the death of PBS-pretreated mice within 72 h (see Fig. 3b), which was associated with extensive hepatocellular damage, as indicated by a massive increase in serum aminotransferases and strong pathological changes indicated by H&E staining (see Fig. 3c and Supplementary Fig. S7b). In contrast to the PBS-pretreated group, Liuweiwuling-pretreated mice survived a lethal dose of TAA without signs of liver injury, showing normal serum aminotransferase levels and normal liver tissue in a dose-dependent manner (see Fig. 3b,c and Supplementary Fig. S7b).

### Validation of the mechanism of Liuweiwuling on liver failure via the inhibition of hepatocyte apoptosis

#### Liuweiwuling pretreatment reduces hepatocyte apoptosis in GalN/LPS- and TAA-treated mice

**Figure 4:**
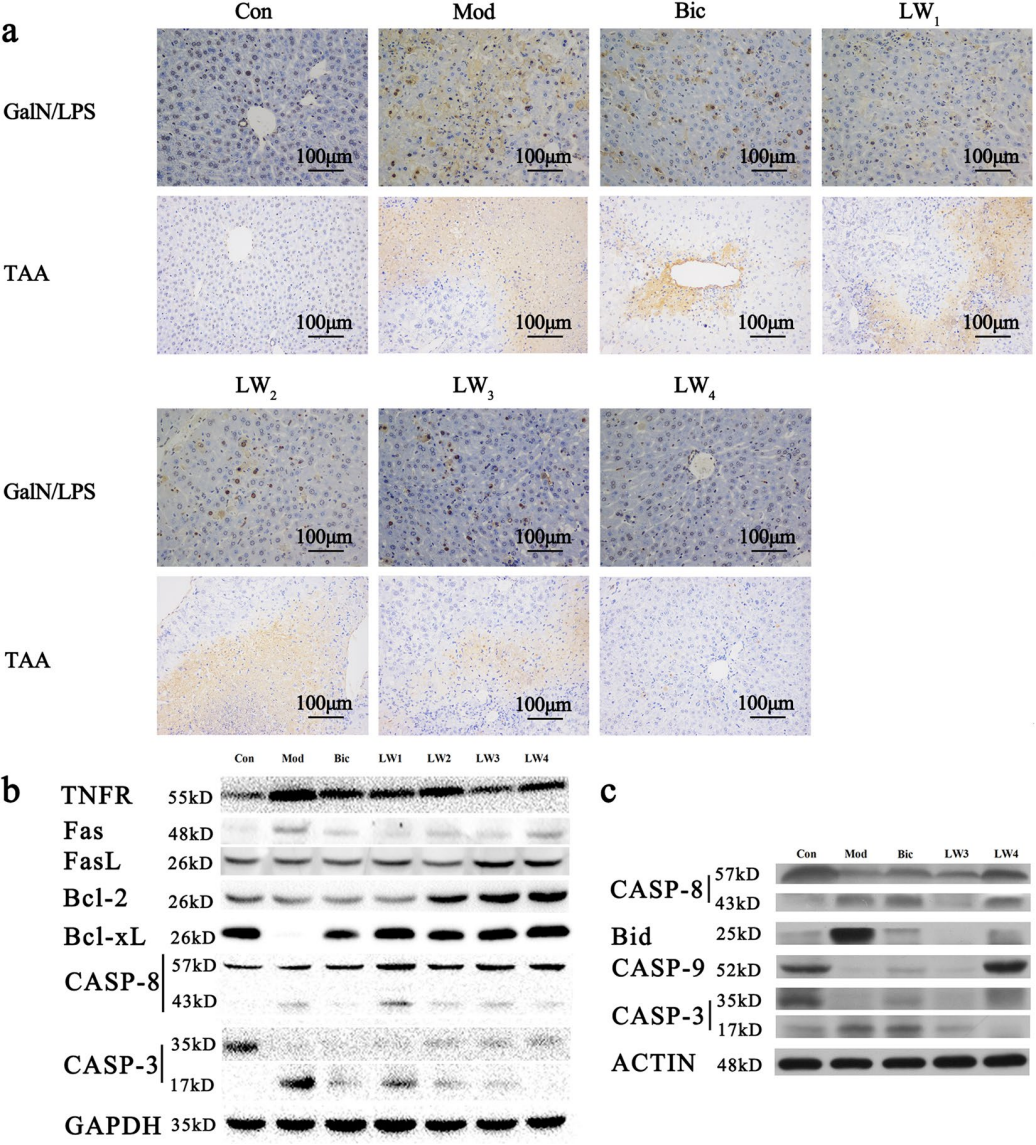
Liuweiwuling abrogates GalN/LPS or TAA-induced ALF by inhibiting both the extrinsic (death receptor pathway) and intrinsic (mitochondrial death pathway) apoptotic pathways. (**a**) A TUNEL assay 6 h after GalN/LPS treatment or 12 h after the last treatment with TAA for the different groups as indicated. In the TUNEL assay, sepia cells indicate apoptotic cells. (**b**) The protein expression of several molecules related to the extrinsic (death receptor pathway) and intrinsic (mitochondrial death pathway) apoptotic pathways in the livers of GalN/LPS-induced mice by western blotting (representative of three independent experiments). (**c**) The protein expression of several molecules related to the extrinsic (death receptor pathway) and intrinsic (mitochondrial death pathway) apoptotic pathways in the livers of TAA-induced mice by western blotting (representative of three independent experiments). For the abbreviation of groups in experimental design, control group (Con), model group (Mod), positive drug group (Bic), 0.5 mg/g Liuweiwuling group (LW1), 2 mg/g Liuweiwuling group (LW2), 8 mg/g Liuweiwuling group (LW3), and 16 mg/g Liuweiwuling group (LW4).

To confirm that Liuweiwuling can suppress hepatocyte apoptosis in GalN/LPS- and TAA-treated mice, we determined the number of apoptotic hepatocytes by the terminal deoxynucleotidyl transferase dUTP nick end labelling (TUNEL) assay. No TUNEL-positive cells were found in the liver tissue specimens of the control group. A large number of apoptotic hepatocytes was observed in the livers of mice 6 h after GalN/LPS injection. The number of TUNEL-positive cells was remarkably higher 12 h after consecutive TAA injections. Hepatocyte apoptosis was inhibited by increasing doses of Liuweiwuling, showing the potent effect of Liuweiwuling on suppressing hepatocyte apoptosis induced by different hepatotoxins (Fig. 4a and Supplementary Fig. S8a,b).

#### Liuweiwuling abrogates hepatocyte apoptosis by suppressing both death receptor and mitochondrial death signalling

**Figure 5:**
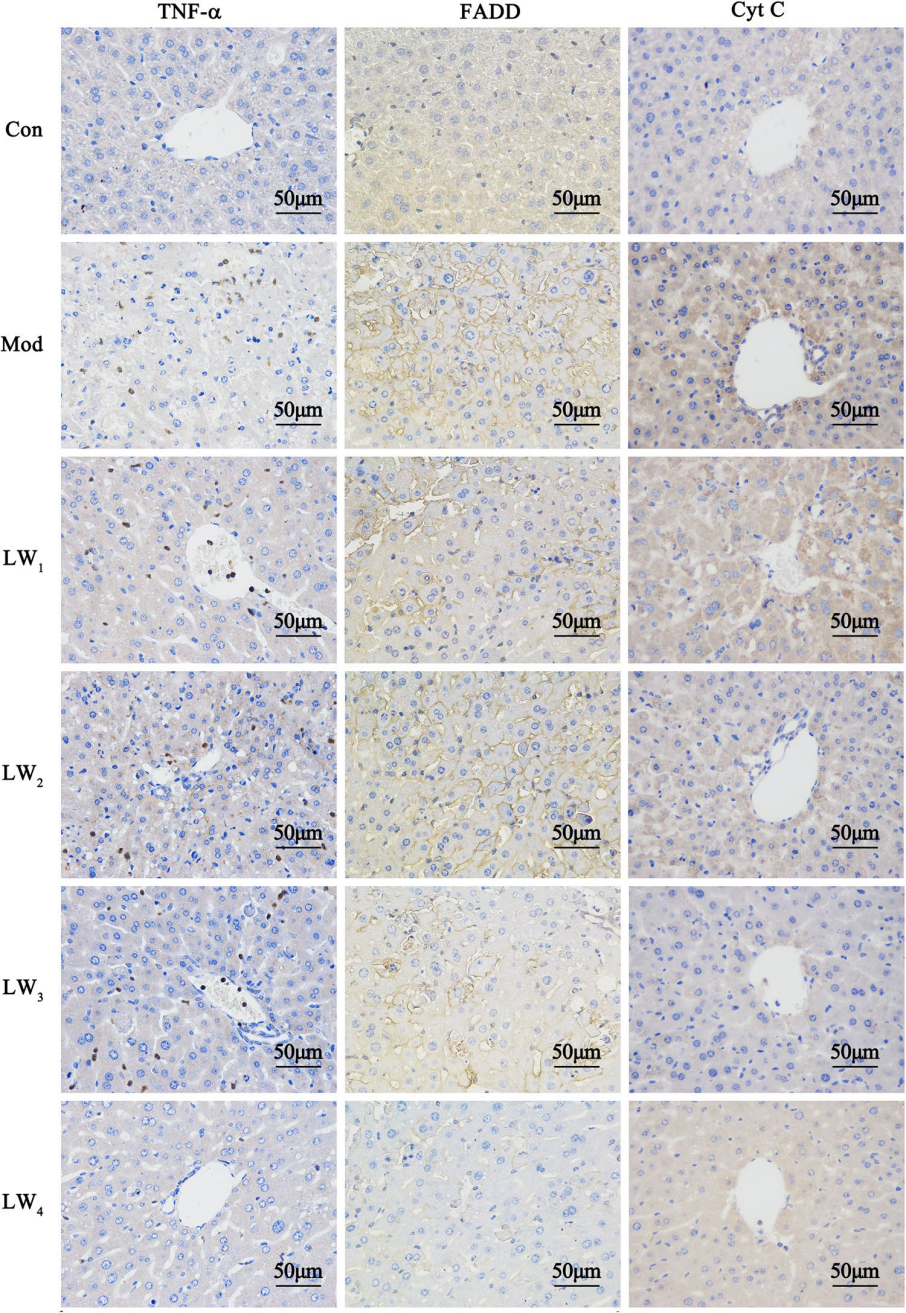
Liuweiwuling administration suppresses TNF-α, FADD and Cyt C protein expression in the hepatocytes of GalN/LPS-treated mice by immunohistochemistry. For the abbreviation of groups in experimental design, control group (Con), model group (Mod), positive drug group (Bic), 0.5 mg/g Liuweiwuling group (LW1), 2 mg/g Liuweiwuling group (LW2), 8 mg/g Liuweiwuling group (LW3), and 16 mg/g Liuweiwuling group (LW4).

We quantitatively analysed the protein expression of several molecules related to extrinsic (death receptor pathway) and intrinsic (mitochondrial death pathway) apoptotic pathways by western blotting (Fig. 4b,c and Supplementary Fig. S9) and immunohistochemistry (see Fig. 5 and Supplementary Fig. S10). The results showed the activation of caspase-8 and caspase-3 in liver homogenates after hepatotoxin injection, as indicated by the detection of specific bands for the caspase-8 subunit (at approximately 43 kDa) and cleaved caspase-3 (at approximately 17–19 kDa). However, Liuweiwuling pretreatment suppressed the activation of caspase-8 and the cleavage of caspase-3. The expression of TNF receptor p55 showed remarkable increases in the livers of GalN/LPS-injected mice, and Liuweiwuling inhibited the expression of these proteins. As well, the results of immunohistochemistry showed the inhibitory effect of Liuweiwuling on TNF-α, Cyt C and FADD in the livers of GalN/LPS-treated mice (Fig. 5). Moreover, the protein expression of Bid (at approximately 20 kDa) was increased in the livers of TAA-injected mice, whereas Liuweiwuling pretreatment significantly suppressed the expression of Bid in the livers of TAA-injected mice. Additionally, the protein expression of Bcl-2 and Bcl-xL (at approximately 25 kDa) was decreased in the livers of GalN/LPS-injected mice, whereas Liuweiwuling pretreatment significantly up-regulated Bcl-2/Bcl-xL protein expression in the livers of GalN/LPS-injected mice.

### Validation of multiple synergistic functions of Liuweiwuling on liver failure

**Figure 6:**
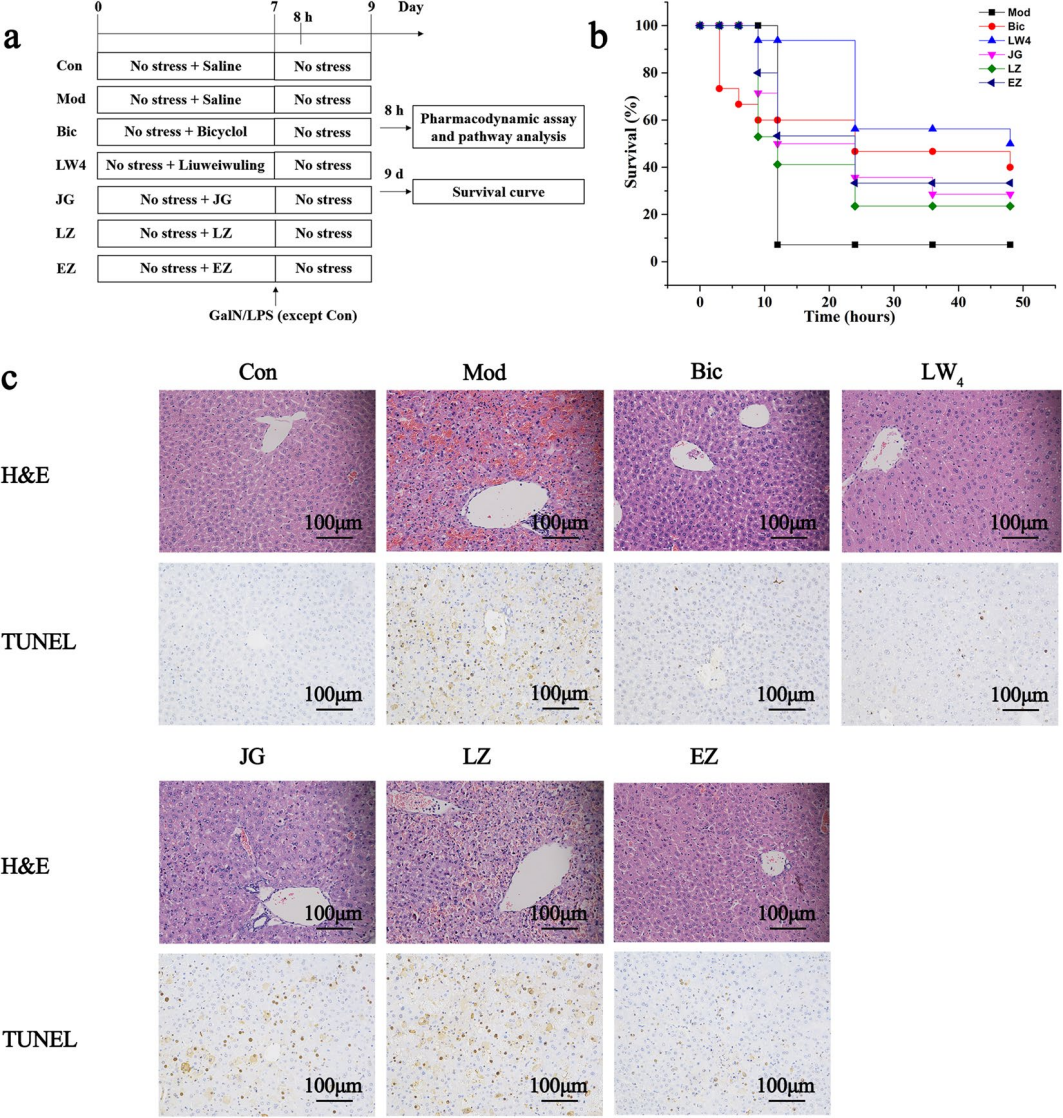
Liuweiwuling and its decomposed ingredients protect against GalN/LPS-induced ALF. (**a**) Experimental design for the present study. (**b**) The survival curves of the different groups (n = 15–20/group) are shown following a GalN/LPS injection. (**c**) H&E staining and a TUNEL assay 6 h after the GalN/LPS treatment of the different groups (n = 6–8/group) as indicated. For the abbreviation of groups in experimental design, control group (Con), model group (Mod), positive drug group (Bic), 16 mg/g Liuweiwuling group (LW4), JG (the mixture of four herbs, *Chinensis* Fructus, *Ligustri Lucidi* Fructus, *Forsythiae* Fructus and *Sonchus brachyotus* DC) group (JG), *Ganoderma* Spore group (LZ), and *Curcumae* Rhizoma group (EZ).

Based on the network results, a decomposition experiment was preliminarily conducted to investigate the synergistic efficacy of the action of Liuweiwuling on liver failure. The experimental design is shown in Fig. 6a. We compared the preventive efficacy of Liuweiwuling with its individual ingredients, JG (the mixture of four herbs, WWZ, NZZ, LQ and BJC in a ratio as 2.34, 1.67, 1, 1, respectively), LZ and EZ, on mortality, the development of hepatic lesions and hepatocyte apoptosis after GalN/LPS injection. First, three ingredients demonstrated similar effects of reducing mortality in GalN/LPS-injected mice, but the effects were less than the effect of Liuweiwuling (see Fig. 6b). In addition, JG had a better protective effect than EZ and LZ in inhibiting the development of hepatic lesions as indicated by ALT and AST activity, the serum level of TNF-α as well as pathological changes indicated by H&E staining. EZ better inhibited hepatocyte apoptosis (see Fig. 6c and Supplementary Figs 11,12) As a result, the preventive efficacy of Liuweiwuling was better than its individual ingredients in all aspects, and this remarkable efficacy was attributed to combined ingredients rather than any certain herb, verifying the synergistic activity of this formula.

## Discussion

Currently, TCM has been confirmed to have significant therapeutic efficacy for complex diseases by exerting pharmacological effects in a multi-component and multi-target manner^22–24^. However, non-specific modern indications and unclear mechanisms due to the differences between *Zheng* and specific diseases restrict the promotion of TCM. In this study, we developed an integrated strategy for the discovery and identification of TCM therapeutic uses by integrating classical pharmacology with systems biology, taking inspiration from drug repositioning methodology^25–27^. We successfully employed this strategy to demonstrate the preventive effect and elementary mechanisms of Liuweiwuling against acute liver failure (ALF). Acute liver failure is a rare but life-threatening critical illness that is typically characterized by massive apoptotic and/or necrotic cell death triggered by activation of tumour necrosis factor (TNF) receptor family members and/or mitochondrial death signalling pathways^25–27^. Currently, more than 50% of acute liver failure cases are related to drug-induced injuries from substances in the US and Europe^28,29^. In China, viral hepatitis, especially hepatitis B, is the most important cause^30^. Emergency liver transplantation is currently considered the only effective treatment for those with advanced disease, but the procedure is limited by a shortage of organ donors and the disadvantage of life-long treatment with immunosuppressants^26,31^. Medical therapies that interrupt the progression of hepatic injury and the development of extrahepatic organ dysfunction are not readily available, making a preventive therapy indispensable^26^. Thus, the discovery of TCM formulae as a complementary and alternative therapy for liver failure is extremely urgent, and the strategy in this paper may provide a reference for these unmet clinical needs.

At present, approaches for discovering new therapeutic uses for TCM formulae in China mainly rely on formulae principal and retrospective analysis. However, this method depends on the scientific support of TCM theory and sufficient clinical sources, which partially limit its application. To address this issue, we have developed a more comprehensive approach that integrates various hepatic disease-drug target networks to effectively discover new therapeutic potentials for Liuweiwuling. Technically, the prediction accuracy of the drug targets and the completeness of the databases, including the drug databases, disease databases, and PPI databases, are critical to this approach and will affect the creditability of the final results. Therefore, we do our utmost to reduce the false positive cases, such as threshold filtering with the fit score in the drug target prediction, significance analysing with hypergeometric distribution method in disease targets enrichment, and reasonable topologic parameter screening in network analysis^25–27^. As a result, liver failure contained more targets and more significant “p-value” than other hepatic diseases. In particular, formulae principal and a retrospective analysis provided a credible reference and indicated that this prediction was credible (see Supplementary Tables S1-S4). Additionally, about the detailed information how to identify 219 compounds from Liuweiwuling and 229 potential target proteins for Liuweiwuling. TCM Database@Taiwan and TCM-SP are all the open databases which provide more details about the chemical components in different herbs, such as chemical names, CAS Nos and chemical structures. When collect 219 compounds from Liuweiwuling, the chemical structure files (*.mol2) of the components in Liuweiwuling were collected from the open online databases including TCM Database@Taiwan (TDT) and TCM-SP databases. For 229 potential target proteins for Liuweiwuling, the putative targets of these compounds might act on were predicted by Pharmmapper via a molecular docking method towards the known human proteins (http://59.78.96.61/pharmmapper/). Only the targets with fit score more than 4.0 were reserved. In summary, the approach based on system biology developed in this paper may provide a new strategy for the more efficient discovery of new therapeutic uses for TCM formulae. Further detailed studies will be performed in our next study.

Subsequently, we validated the efficacy of Liuweiwuling in protecting mice against two classical toxicant-induced death; thus demonstrating the efficacy of Liuweiwuling in preventive settings. The administration of a subtoxic dose of D-galactosamine (GalN) with lipopolysaccharide (LPS) causes hepatic damage through the over-production of secreted TNF-α, leading to massive hepatocyte apoptosis, and it has often been used as an animal model of ALF to decipher mechanisms and find potential therapies^32–38^. Thioacetamide (TAA) is a classic hepatotoxin known to induce highly reproducible ALF, which predominantly causes acute centrilobular necrosis due to dramatically increased production of reactive oxygen species^38–44^. Our results show that Liuweiwuling has a therapeutic effect by blocking hepatocyte apoptosis in a highly efficient manner, more than a TNF-antagonist. Then, we verified the multi-target efficacy of the inhibition of liver apoptosis by analysing the protein expression of several molecules related to multiple apoptotic pathways. Apoptosis can be initiated by two fundamental extrinsic or intrinsic pathways^45^. Extrinsic apoptosis is mediated by death receptors, a subfamily of the TNF receptor superfamily, including TNFR1 and FAS, which lead to the formation of a DISC (death-inducing signalling complex) that activates caspase-8 and drives the activation of effector caspases^46,47^. The intrinsic pathway of apoptotic cell death depends on permeabilization of the mitochondrial outer membrane regulated by pro-apoptotic and anti-apoptotic members of the Bcl-2 protein family, which relies on tBid generation to trigger the mitochondrial cell death pathway^47,48^. Following mitochondrial permeabilization, pro-apoptotic factors, such as cytochrome c, are released into the cytosol, triggering the activation of caspase-9 and downstream effector caspases^46,49^. Our results showed that Liuweiwuling might provide a systematic therapy for apoptosis more than a single central death pathway. Taken together, based on the crucial role of hepatocyte apoptosis in a broad spectrum of acute and chronic liver disease^50,51^, Liuweiwuling could be of therapeutic benefit for preventing and treating liver injuries because of its easy application through an anti-apoptotic approach. Additionally, Liuweiwuling has shown the efficacy of promoting cell proliferation for treating liver failure (see Supplementary Fig. S28), and we may further discuss the specific mechanism of Liuweiwuling on liver failure by promoting cell proliferation in our next mechanism-exploring paper.

**Figure 7:**
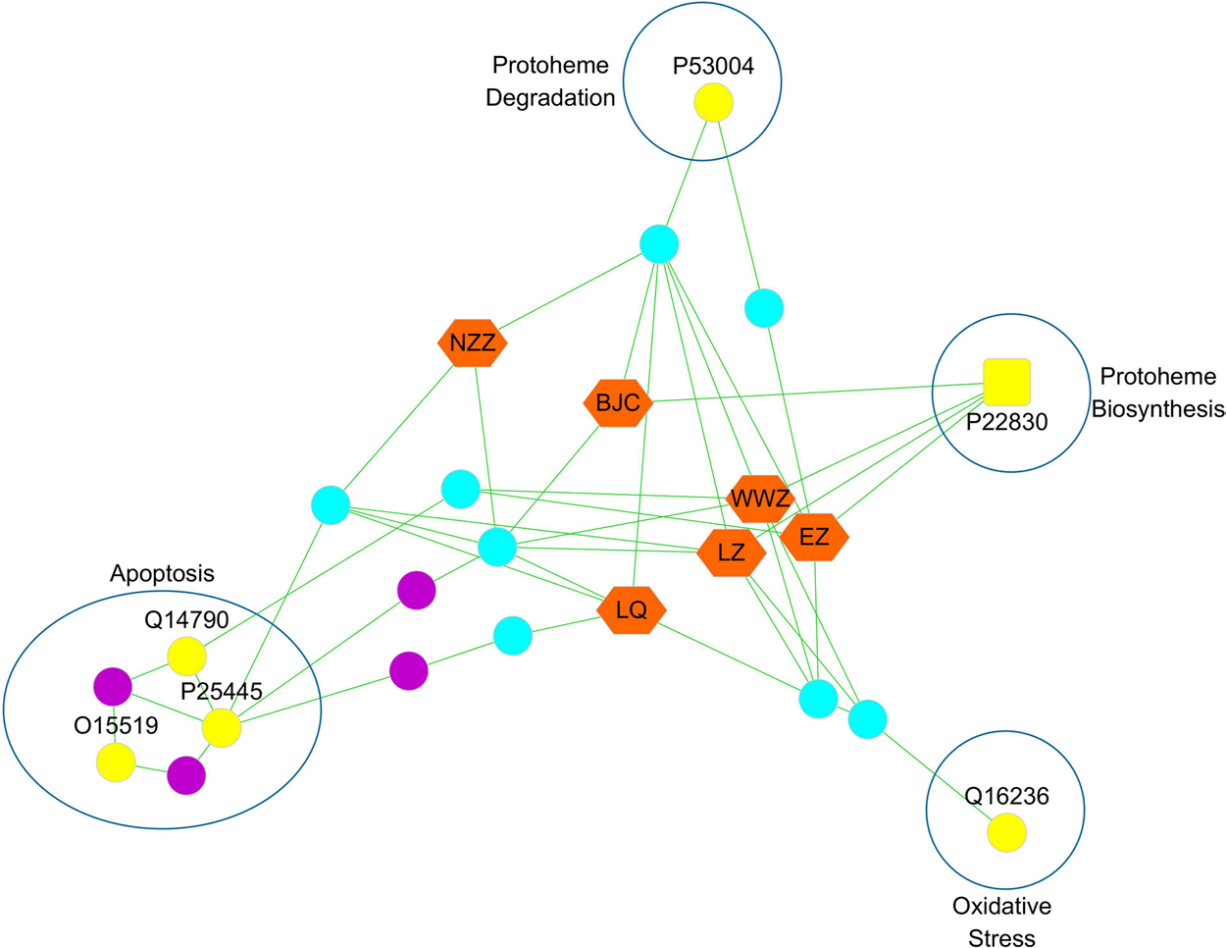
The major actions of each composed herb in Liuweiwuling working synergistically on the treatment of liver failure. Notes: The orange hexagon nodes are the six herbs that constitute Liuweiwuling: *Schisandrae Chinensis* Fructus (WWZ), *Ligustri Lucidi* Fructus (NZZ), *Forsythiae* Fructus (LQ), *Sonchus brachyotus* DC. (BJC), *Curcumae* Rhizoma (EZ) and *Ganoderma* Spore (LZ).The azure dots represent the indirect targets for drugs; the yellow dots represent the targets of the specific disease of liver failure; the yellow squares represent the common targets of herbs and liver failure; the purple dots represent the interactional proteins with the targets of liver failure and the drugs.

Finally, we predicted the main efficacy of each herb in the mixture by simplifying the herb-chemical component candidate drug target and the major liver failure target network. Based on an overview of essential liver failure targets with distinct functions^52–56^, we analysed the different processes by which the individual Liuweiwuling herbs act on liver failure. According to our results, EZ affects apoptosis, jaundice and proliferation, especially a direct effect on caspase-3. LZ affects oxidative damage and the immune system. BJC, NZZ and LQ similarly affect immune-mediated apoptosis. WWZ affects almost all of the above aspects of the other individual herbs on liver failure. Although network pharmacology provides a rough direction, substantial experimental data are necessary for verification. We compared the preventive effect of EZ, LZ and JG (BJC, NZZ, LQ, and WWZ) on liver failure with Liuweiwuling through a preliminary decomposition experiment, and the results partially agreed with the network analysis described above, especially the anti-apoptotic effect of EZ. Although the effect of EZ on the activity of serum aminotransferase and serum TNF-α was lower than that of JG, EZ more effectively reduced mortality and inhibited apoptosis in GalN/LPS-injected mice, verifying the important role of apoptosis in Liuweiwuling action on liver failure. However, the preventive effect of Liuweiwuling was better than that of its constituents in all aspects, verifying that it provides a synergistic therapy for this complex disease. Additionally, in view of network analysis, the major actions of each composed herb in Liuweiwuling working synergistically on the treatment of liver failure were shown as Fig. 7, which deserves further research and we may design the next experiment about this.

There remains a long way to go before the therapy and theory of TCM/formulae can be recognized as a science, and we should strive unremittingly to convert its traditional efficacy into modern therapeutic value. To reach this goal, further development and incorporation of many disciplines (such as biochemistry, molecular biology and bioinformatics) are necessary for the elucidation of the curative and biological mechanisms of TCM/formulae. The strategy we propose here provides a new way to identify modern indications instead of *Zheng* for TCM/formulae with notable clinical efficacy by an integrated approach. We hope this work will make a contribution to the development of future medicines based on the wisdom of Chinese medicine for unmet clinical needs.

## Materials and Methods

### Quality control of Liuweiwuling

The quality of Liuweiwuling for animal studies was ensured by the establishment of a chemical fingerprint. Full methods and any associated operational details are available in the supplementary materials^57^.

### Database construction

For the disease target database construction, 749 targets related to 63 liver diseases were included by keyword-based searching over the Online Mendelian Inheritance in Man (OMIM) database, and the disease names and keywords were refer to the Medical Subject Headings (MeSH) database, NIH U.S. National Library of Medicine. For the drug target database construction, the chemical structure files (*.mol2) of the components in Liuweiwuling were collected from the open online databases including TCM Database@Taiwan (TDT) and TCM-SP databases, and the putative targets of these compounds might act on were predicted by Pharmmapper via a molecular docking method towords the known human proteins (http://59.78.96.61/pharmmapper/). Only the targets with fit score more than 4.0 were reserved. All of the targets were described as their uniprot IDs^14^.

### Network construction and analysis

Based on^11^ the databases above mentioned, a disease specified network was constructed as following: Firstly, the drug targets were connected to the drugs components by the prediction information from Pharmmapper, threshold filtering with the fit score (Score ≥ 4.0) in the drug target prediction by network analysis; Secondly, the drug targets and the liver disease targets were connected with protein-protein interactions in the Database of Interacting Proteins (DIP) database, and the disease targets that can’t be connected were discarded; Thirdly, the drug components, putative drug targets, disease-significant targets and interactional proteins were combined to construct a “compound–target–disease” network. To discover the primary therapeutic uses of Liuweiwuling, an enrichment analysis with hypergeometric distribution was applied to the rest disease targets against the disease targets database, which was referred to some enrichment analysis methods in the Gene Ontology (GO) database^15–17^. The major predicted diseases that Liuweiwuling might act on were indicated with a hypergeometric p-value less than 0.05. Then, the disease specified network of the selected disease was rebuild and Cytoscape V3.2 was applied to visualize and analyse the network. The topological features of each node in the network were calculated using NetworkAnalyzer in the Cytoscape software and the most possible diseases targets that Liuweiwuling might act on were screened with three topologic parameters: “Degree”, “Betweenness Centrality” and “Closeness Centrality”. Only the hub nodes (“Degree” values 2-fold greater than the median value of all the network nodes) with higher values of “Betweenness centrality” and “Closeness centrality” (above the median value of all the network nodes) were identified as the candidate Liuweiwuling targets.

### Drugs and reagents

Liuweiwuling (Shibojindu Pharmaceutical Company, Shandong, China, Batch No.: 141203, 141205, 150509, 151105) and bicyclol (Beijing Union Pharmaceutical Factory, Beijing, China, Batch No.: 141203) were obtained from the 302 Military Hospital of China Pharmacy, Beijing, China. JG (Batch No.: 151002), EZ (Batch No.: 150902) and LZ (Batch No.: 150912) were obtained from Shandong Shibojindu Pharmaceutical Company, Shandong, China. Lipopolysaccharide (LPS) from *E. coli* 055: B5, D-galactosamine (GalN) and thioacetamide (TAA) was purchased from Sigma-Aldrich, St. Louis, MO, USA. Both reagents were AR grade. Alanine aminotransferase (ALT) and aspartate aminotransferase (AST) test kits were purchased from Nanjing Jiancheng, Nanjing, China. An *In Situ* Cell Death Detection Kit was purchased from Roche Life Science, Indianapolis, IN, USA. TNF-α antibody were purchased from Wuhan Gugebio, Wuhan, China. Cyt C, FADD and caspase-9 antibodies were purchased from Abcam, Cambridge, UK. TNFR, Bcl-2, Bcl-x_L,_ FasL and Fas antibodies were purchased from Santa Cruz Biotechnology, Santa Cruz, CA, USA. Caspase-3, GAPDH and HRP-conjugated anti-rabbit IgG antibodies were purchased from Cell Signaling Technology, Danvers, MA, USA. Bid and caspase-8 were purchased from R&D Systems, Minneapolis, MN, USA. HRP-conjugated anti-mouse IgG was purchased from Santa Cruz. The EasySee Western Blot Kit was purchased from TransGen Biotech, Beijing, China.

### Animals

Male BALB/c mice and female C57BL/6 mice, aged 6–8 weeks and weighing 20 ± 2 mg, were purchased from the Laboratory Animal Centre of the Academy of Military Medical Sciences (certification number SYXK-JUN 2012–0010). All cages contained wood-chips and were placed in a temperature-controlled room at 20 ± 2 °C with 60–70% humidity. A 12-h light/dark cycle was set, and the animals were provided free access to a standard diet and water. The animals were acclimated for 7 d prior to the experiments. For survival experiments, the animals were euthanized when they became moribund according to the criteria of lack of response to stimuli or lack of a righting reflex. All animal protocols were approved by the Committee on the Ethics of Animal Experiments of the 302 Military Hospital (Approval ID: IACUC-2015-022).

### GalN/LPS- and TAA-induced hepatotoxicity

A GalN/LPS solution was made fresh for each experiment in pyrogen-free PBS and injected intraperitoneally. GalN was dosed at 800 mg/kg, and LPS was dosed at 10 μg/kg. Control mice received an appropriate volume of pyrogen-free PBS. Animals were euthanized by a ketamine/xylazine injection at 6 h for collection of serum and liver tissue. For survival experiments, the animals were observed for 48 h. A TAA solution was made fresh for each experiment in pyrogen-free PBS at 20 mg/ml. TAA was dosed at 750 mg/kg, depending on the experiment, and injected intraperitoneally on two consecutive days. Control mice received an appropriate volume of pyrogen-free PBS. The animals were euthanized by ketamine/xylazine injection at 12 h after the last dose of TAA for the collection of serum and liver tissue. For survival experiments, animals were observed for 72 h. Bicyclol was used as a positive control, the dose of bicyclol is 9.75 g/g^58^.

### ALT, AST and histology

Serum samples were obtained by separating the supernatant from the blood after 30 min of coagulation at room temperature. After centrifugation (3,000 rpm for 10 min), the serum ALT and AST levels were measured with an Olympus AU5400 automatic biochemistry analyser (Olympus Optical, Tokyo, Japan). Liver tissue was fixed and preserved in 10% neutral buffered formalin for 2 days, embedded in paraffin and processed for sectioning to a thickness of approximately 5 μm for histopathological examination. For histological staining, paraffin-embedded sections of liver tissue were stained with H&E.

### Apoptosis

Liver cell apoptosis was assessed by the *in situ* terminal deoxynucleotidyl transferase-mediated dUTP nick-end labelling (TUNEL) assay with an *In Situ* Cell Death Detection Kit according to Gavrieli *et al*.^59^ Briefly, sections were treated with proteinase K, and endogenous peroxidase activity was blocked by treatment with 0.02% hydrogen peroxide. Tissue sections were treated with a mixture of terminal deoxynucleotidyl transferase (Wuhan Gugebio Co., Ltd), digoxigenin-labeled dUTP and dATP at 37 °C for 1 h followed by incubation with peroxidase-labelled anti-digoxigenin antibody (Wuhan Gugebio Co., Ltd) solution for 30 min. The reaction products were revealed using nickel and cobalt-3, 3′-diaminobenzidine (EnVision Detection Systems Peroxidase/DAB, Rabbit/Mouse; DAKO Agilent Pathology Solutions Co., Ltd, k5007), and tissue sections were counterstained with haematoxylin (Wuhan Gugebio Co., Ltd, G1004).

### Western blotting

The liver samples were homogenized in lysis buffer [1% Triton X-100, 1% sodium deoxycholate and 0.1% sodium dodecyl sulfate (SDS)], incubated on ice for 30 min and centrifuged at 12,000 r/min at 4 °C for 10 min for collection of the supernatants. The protein concentration of the supernatant was determined with a BCA protein assay kit and then boiled for 10 min. Equal amounts of protein were loaded onto a 15% SDS-polyacrylamide gel for electrophoresis (PAGE) and transferred to a polyvinylidene fluoride (PVDF) membrane. Then, the PVDF membrane was blocked with blocking buffer containing 5% non-fat milk in 0.1% Tris-buffered-saline tween 20 (TBST) for 2 h at room temperature followed by overnight incubation at 4 °C with specific primary antibodies against TNFR (1:1000), Fas (1:200), FasL (1:2000), Bid (1:1000), caspase-9 (1:1000), Bcl-2 (1:1000), Bcl-x_L_ (1:800), caspase-8 (1:500) and caspase-3 (1:1000). GAPDH (1:1000) was used as a loading control. After three 5-min washes with TBST (5 × TBS, 0.05% Tween 20), the PVDF membranes were incubated with secondary horseradish peroxidase (HRP)-conjugated IgG antibody (1:3000) for another 1 h at room temperature. The PVDF membranes were treated with an EasySee Western Blot Kit and then exposed to the Tanon 4200SF Chemiluminescent Imaging System (Shanghai Tanon Science & Technology Co., Ltd.).

### Immunohistochemistry of TNF-α, FADD and Cyt C

Immunohistochemistry^60^ for TNF-α, Cyt C and FADD was performed with anti-TNF-α antibody, anti-Cyt C antibody and anti-FADD antibody, respectively (Wuhan Gugebio, GB13188; Abcam, ab133504; Abcam, ab124812). Paraffin-embedded liver sections were dewaxed and retrieved in EDTA buffer (PH 8.0) via microwave antigen retrieval. The sections were treated with 3% H2O2 in PBS for 25 min, washed twice in PBS and then blocked in 3% bovine serum albumin in PBS for 30 min at room temperature. The sections were incubated with anti-TNF-α antibody, anti-Cyt C antibody and FADD antibody diluted 1:100 in PBS containing 1% bovine serum albumin at 4°C overnight, followed by washing twice in PBS. The sections were incubated with an HRP-labelled secondary antibody (KPL, Baltimore, Maryland, USA 074-1506) for 50 min at room temperature. The reaction was visualized with DAB solution (Dako, Carpinteria, CA) after washing twice in PBS and then counterstained with Mayer’s haematoxylin.

### Statistical analysis

The results are expressed as the means plus/minus the standard deviations. The data were analysed with the SPSS software program (version 22.0, Chicago, IL, USA). Statistical comparisons were performed using a one-way analysis of variance (ANOVA) with a post hoc test followed by Student’s t-test (the Mann-Whitney U test was used when the t-test was not suitable). For the evaluation of significant differences, *P* < 0.05 was considered significant, and *P* < 0.01 was considered highly significant.

### Ethics statement

This study presented in the manuscript involve animal subjects. The full name of the ethics committee that approved the study is Institutional Animal Care and Use Committee of 302 hospital of PLA. This study was conducted in strict accordance with the recommendation of the Guidelines for the Care and Use of Laboratory Animals of the Ministry of Science and Technology of China. No vulnerable populations, persons with disabilities or endangered animal species were used in this study.

## Electronic supplementary material

Supplementary information
