# Supplementary material for: A systems pharmacology-oriented discovery of a new therapeutic use of the TCM formula Liuweiwuling for liver failure
Source: Sci Rep. 2018 Apr 4;8:5645. doi: 10.1038/s41598-018-21515-6 (PMC5884779; doi:10.1038/s41598-018-21515-6)
Supplement: Supplementary file 1 — Supplementary information [file 41598_2018_21515_MOESM1_ESM.docx]

***Supplementary Information for***

**A systems pharmacology-oriented discovery of a new therapeutic use of the TCM formula Liuweiwuling for liver failure**

Jia-bo Wang^1‡^, He-rong Cui^1,2‡^, Rui-lin Wang^1,3‡^, Cong-en Zhang^1,4‡^, Ming Niu^1*^, Zhao-fang Bai^1*^, Gen-hua Xu^1^, Peng-yan Li^1^, Wen-yan Jiang^1^, Jing-jing Han^1^, Xiao Ma^4^, Guang-ming Cai^1^, Rui-sheng Li^5^, Li-ping Zhang^2^, Xiao-he Xiao^1, 3*^

*^1^ China Military Institute of Chinese Medicine, 302 Military Hospital, Beijing, PR China.*

*^2^ Traditional Chinese Medicine College, Beijing University of Traditional Chinese Medicine, Beijing, PR China.*

*^3^ Integrative Medicine Center, 302 Military Hospital, Beijing, PR China.*

*^4^ College of Pharmacy, Chengdu University of Traditional Chinese Medicine, Chengdu, PR China.*

*^5^ Research Center for Clinical and Translational Medicine, 302 Hospital of PLA, Beijing, PR China.*

Correspondence:

Ming Niu, Zhao-fang Bai, and Xiao-he Xiao

302 Military Hospital, No. 100 Xisihuan Beijing 100039, China

Fax: +86 66933322; +86 66933325

E-mail: nmbright@163.com; pharm_sci@126.com; pharmacy302xxh@126.com

‡ These authors contributed equally to this work.

* To whom correspondence should be addressed.

**A list of figures and tables in the Supplementary Information**

| No. | Headline | Figures/Tables |
| --- | --- | --- |
| 1 | *Zheng* disease analysis of Liuweiwuling and liver failure | Table S1-S2 |
| 2 | The preventive effect of Liuweiwuling on liver failure by a retrospective analysis based on small sample data | Table S3-S4 |
| 3 | Component identification of Liuweiwuling by LC-QTOF/MS | Figure S1;  Table S5 |
| 4 | Multicomponent quantification of Liuweiwuling by HPLC | Figure S2;  Table S6 |
| 5 | HPLC fingerprint analysis of Liuweiwuling | Figure S3;  Table S7-S8 |
| 6 | Prediction of the underlying mechanisms of Liuweiwuling acting on liver failure by network pharmacology | Figure S4 |
| 7 | Major candidate drug targets of Liuweiwuling for treating liver failure | Table S9 |
| 8 | Major liver failure targets associated with the main efficacy of each individual herb in Liuweiwuling | Table S10 |
| 9 | The protein expression and the mRNA levels of major candidate drug targets of Liuweiwuling for treating liver failure | Figure S5-S6;  Table S11 |
| 10 | Serum aminotransferase levels (alanine aminotransferase [ALT] and aspartate aminotransferase [AST]) following GalN/LPS or TAA injection | Figure S7 |
| 11 | A TUNEL assay 6 h after GalN/LPS treatment or 12 h after the last treatment with TAA for the different groups | Figure S8 |
| 12 | The greyscale of western blot results in the apoptosis pathways | Figure S9 |
| 13 | Liuweiwuling administration suppresses TNF-α, FADD and Cyt C protein expression in the hepatocytes of GalN/LPS-treated mice by immunohistochemistry | Figure S10 |
| 14 | Liuweiwuling and its decomposed ingredients protect against GalN/LPS-induced ALF | Figure S11 |
| 15 | The protective effect of Liuweiwuling and its separate ingredients on serum TNF-α in GalN/LPS-injected mice | Figure S12 |
| 16 | Macroscopic appearance and H&E staining of all liver samples 6 h after GalN/LPS or 12 h after TAA treatment of respective groups in the experiment | Figure S13-S26 |
| 17 | The efficacy of Liuweiwuling on promoting cell proliferation for treating liver failure by immunohistochemistry | Figure S27 |
| 18 | All drug targets and diseases targets associated with Liuweiwuling or liver failure | Table S12-S13 |

- ***Zheng* disease analysis of Liuweiwuling and liver failure**

We analysed the TCM theory and formulae principal of Liuweiwuling based on The Foundations of Chinese Medicine, The Yellow Emperor’s Classic of Medicine and Herbal Prescription Science. According to TCM theory and the formulae principle, the appropriate *Zheng* of Liuweiwuling is associated with clinical pathogenesis, including poison, dampness, heat, stasis and deficiency in liver or kidney, which is in accordance with the TCM therapeutic methods and principles for different categories of liver failure (see Supplementary Table S1-S2).

| **Table S1** \| Formulae principal and appropriate *Zheng* of Liuweiwuling | | | |
| --- | --- | --- | --- |
| Composition of Liuweiwuling | Formulae principal | Efficacy of combination | Appropriate *Zheng* with pathogenesis |
| *Schisandrae Chinensis* Fructus | Enhancing Qi while nourishing fluid | Tonifying Qi and nourishing Yin, clearing heat and removing dampness, removing heat to cool blood and resolving stasis | Poison, dampness, heat, stasis and deficiency, especially liver and kidney associated diseases |
| *Ligustri Lucidi* Fructus | Nourishing liver and kidney |  |  |
| *Forsythiae* Fructus | Clearing away heat and toxicity |  |  |
| *Sonchus brachyotus* DC. | Clearing away heat and toxicity |  |  |
| *Curcumae* Rhizoma | Activating blood stagnation and resolving stasis |  |  |
| *Ganoderma* Spore | Increasing organism immunity and supporting healthy energy |  |  |

| **Table S2** \| TCM therapeutic methods and principles for different categories of liver failure | | |
| --- | --- | --- |
| Clinical classification | Pathogenesis | Therapeutic methods and principles |
| Acute liver failure | Poison, dampness, heat | Clearing heat and removing dampness |
| Acute-on-chronic liver failure | Poison, heat, deficiency | Clearing away heat and toxicity |
| Chronic liver failure | Deficiency, dampness, heat, stasis | Enhancing the Yang and clearing away heat |

- **The preventive effect of Liuweiwuling on liver failure by a retrospective analysis based on small sample data**

Seventy-two cases with a history of chronic hepatitis B infection were retrospectively collected from 783 patients hospitalized in 20 departments of the 302 Military Hospital. All the enrolled patients had a history of chronic hepatitis B infection; Liuweiwuling was used in 30 cases, whereas 42 cases had not received Liuweiwuling. Compared with the Liuweiwuling-untreated cases, the cases treated with Liuweiwuling showed considerable differences in the occurrence of liver failure, and the results are shown as Supplementary Table S3-S4.

| **Table S3** \| Liuweiwuling-treated and liver failure occurrence cross-tabulation | | | | |
| --- | --- | --- | --- | --- |
|  | | Liver failure occurrence | | Total |
|  |  | No | Yes |  |
| Liuweiwuling-treated | Yes | 24 | 6 | 30 |
|  | No | 20 | 22 | 42 |
| Total | | 44 | 28 | 72 |

| **Table S4** \| Chi-square tests for Liuweiwuling-treated and liver failure occurrence cross-tabulation | | | | | |
| --- | --- | --- | --- | --- | --- |
|  | Value | df | Asymp. Sig. (2-sided) | Exact Sig. (2-sided) | Exact Sig. (1-sided) |
| Pearson Chi-Square | 7.721^a^ | 1 | 0.005 |  |  |
| Continuity Correction^b^ | 6.419 | 1 | 0.011 |  |  |
| Likelihood Ratio | 8.075 | 1 | 0.004 |  |  |
| Fisher's Exact Test |  |  |  | 0.007 | 0.005 |
| Linear-by-Linear Association | 7.614 | 1 | 0.006 |  |  |
| N of Valid Cases | 72 |  |  |  |  |
| a. No cells (0.0%) had an expected count less than 5. The minimum expected count was 11.67. | | | | | |
| b. Computed only for a 2x2 table. | | | | | |

- **Component identification of Liuweiwuling by LC-QTOF/MS**

An LC–MS analysis was performed using an Agilent 1290 series UHPLC system coupled to a 6550 Q-TOF/MS mass spectrometer (Agilent Technologies, USA). The analysis was conducted on a ZORBAX RRHD 300 SB-C18 column (2.1×100 mm, 1.7 μm i.d., Agilent Technologies, USA). For the ESI+ analysis, the mobile phases were solvent A (Water) and solvent B (Acetonitrile) with a gradient elution as follows: 10% – 23% B at 0 – 7 min, 23% B at 7 – 12.5 min, 23% – 40% B at 12.5 – 20 min, 40% - 70% B at 20 – 40 min, 70% - 100% B at 40 – 45 min. The flow rate was 0.300 mL/min. The column and autosampler were maintained at 30ºC and 4°C, respectively. The injection volume of the samples was 1 μL. For the ESI- analysis, the mobile phases were solvent A (CH_3_OH) and solvent B (H_2_O), and the other analysis conditions were identical to the ESI+ analysis.

A high-resolution electrospray mass spectrometer was operated in a negative (ESI-) ion mode for LC-MS analysis. For the full-scan MS analysis, the spectrum was recorded in a range of m/z 100-1000. The optimal conditions of analysis were as follows: the capillary voltage was 4 kV, the desolvation gas flow was 11.0 L/min, the source temperature was set at 125ºC and the desolvation gas temperature was 225ºC. Calibrations were automatically conducted from m/z 100 to 1000 with a solution of sodium formate.

To identify the principal components in Liuweiwuling, the chemical constituents were tentatively identified based on accurate mass and a reference to the related literature. Six compounds were tentatively identified, including 6 common peaks in the ESI+ analysis and 3 common peaks in the ESI- analysis. The mass data and compounds identified from the peaks are summarized in Supplementary Fig. S1 and Table S5.

**Figure S1** | **A base peak chromatogram of the LC-MS analysis of Liuweiwuling in the ESI+ mode (a) and ESI- mode (b).**

| **Table S5** \| Identification of components contained in Liuweiwuling | | | | | | | | | | | |
| --- | --- | --- | --- | --- | --- | --- | --- | --- | --- | --- | --- |
| No | Positive mode | | |  | Negative mode | | | M(g/mol) | Formula | InChI Key | Chemical Name |
|  | tR(min) | Theoretical value | Actual value |  | tR(min) | Theoretical value | Actual value |  |  |  |  |
| 1 | 1.9000 | 323.1101 | 323.1114 |  | 1.9910 | 335.0903 | 335.0919 | 300.3044 | C_14_H_20_O_7_ | ILRCGYURZSFMEG-RKQHYHRCSA-N | Salidroside |
| 2 | 9.9570 | 557.1993 | 557.2554 |  | 10.0150 | 569.1795 | 569.1775 | 534.5523 | C_27_H_34_O_11_ | KFFCKOBAHMGTMW-LGQRSHAYSA-N | Phillyrin |
| 3 | 21.8130 | 455.2040 | 445.2083 |  | - | - | - | 432.5067 | C_24_H_32_O_7_ | YEFOAORQXAOVJQ-UHFFFAOYSA-N | Schisandrol A |
| 4 | 29.7600 | 559.1939 | 559.2000 |  | 2.5000 | 571.1740 | 571.1668 | 536.5697 | C_30_H_32_O_9_ | UFCGDBKFOKKVAC-DSASHONVSA-N | Schizandrol A |
| 5 | 34.1510 | 417.2272 | 417.2444 |  | - | - | - | 416.5073 | C_24_H_32_O_6_ | JEJFTTRHGBKKEI-OKILXGFUSA-N | Schizandrin A |
| 6 | 36.5870 | 401.1959 | 401.2119 |  | - | - | - | 400.4648 | C_23_H_28_O_6_ | RTZKSTLPRTWFEV-UHFFFAOYSA-N | Schizandrin B |

- **Multicomponent quantification of Liuweiwuling by HPLC**

Analyses were performed using an Agilent 1260 HPLC system (Agilent Technologies, Palo Alto, CA, USA). Chromatography was carried out at 30ºC on a Kromasil 100-5 C18 column (250 mm × 4.6 mm, with 5 μm particle size, AKZO NOBEL, Gothenburg, Sweden). The mobile phase consisted of (A) acetonitrile and (B) 0.01% (v/v) phosphoric acid water solution. The gradient elution was as follows: 5% A from 0 to 5 min, 5 - 18% A from 5 to 20 min, 18% A from 20 to 28.5 min, 18 - 50% A from 28.5 to 50 min, 50 - 55% A from 50 to 57 min, 55% B from 57 to 67 min, 55 - 75% A from 67 to 72 min, 75 - 85% A from 72 to 80 min and 85 - 5% A from 80 to 90 min at a flow rate of 1.0 mL⋅min^−1^. The signal was monitored at 275 nm.

Standards of salidroside, phillyrin, schisandrol A, schizandrol A, schizandrin A and schizandrin B were purchased from the Chengdu Pufei De Biotech, Ltd (Chengdu, Sichuan, China), and the purity of all compounds was higher than 98.0%. HPLC-grade acetonitrile was purchased from Burdick & Jackson (Ulsan, Korea). Water was purified by a Milli-Q Plus water purification system (Millipore, USA). AR-grade phosphoric acid was obtained from Xilong Chemical Co., Ltd. (Beijing, China).

Supplementary Fig. S2a shows a chromatogram of a salidroside, phillyrin, schisandrol A, schizandrol A, schizandrin A and schizandrin B standard mixture. Fig. S2b shows the HPLC profile of Liuweiwuling. The percentages of each compound in Liuweiwuling were calculated by normalization method. As a result, the average concentration of salidroside, phillyrin, schisandrol A, schizandrol A, schizandrin A and schizandrin B in 4 batches of Liuweiwuling was 0.23%, 0.14%, 0.40%, 0.25%, 0.34% and 0.13%, respectively, which was in accordance with the quality standard (YBZ06972006-2009Z, the content of schizandrin A is no less than 1.0 mg / 500 mg) of the China National Drug Standards (2010) (Supplementary Table S6).

**Figure S2** | **Multicomponent quantification of Liuweiwuling.** **a** Chromatogram of salidroside, phillyrin, schisandrol A, schizandrol A, schizandrin A and schizandrin B standard mixture; peak (a) for salidroside; peak (b) for phillyrin; peak (c) for schisandrol A; peak (d) for schizandrol A; peak (e) for schizandrin A and peak (f) for schizandrin B. **b** HPLC profile of Liuweiwuling.

| **Table S6** \| Contents of the components in 4 batches of Liuweiwuling (x ̅± s, n = 6) | | | | |
| --- | --- | --- | --- | --- |
| Constituents (mg/g) | No. 1 | No. 2 | No. 3 | No. 4 |
| Salidroside | 2.15±0.01 | 2.11±0.02 | 2.49±0.02 | 2.28±0.04 |
| Phillyrin | 1.38±0.05 | 1.39±0.10 | 1.44±0.12 | 1.34±0.01 |
| Schisandrol A | 4.07±0.01 | 4.04±0.01 | 3.55±0.02 | 4.24±0.02 |
| Schizandrol A | 2.76±0.06 | 2.42±0.02 | 2.60±0.01 | 2.32±0.01 |
| Schizandrin A | 3.41±0.01 | 3.57±0.02 | 3.43±0.02 | 3.25±0.02 |
| Schizandrin B | 1.24±0.01 | 1.21±0.03 | 1.76±0.01 | 1.11±0.01 |

- **The analysis of fingerprint components in the 4 batches of Liuweiwuling for the multicomponent quantification and quality.**

This analysis was performed using an Agilent 1260 HPLC system. Chromatography was carried out at 30ºC on a Kromasill 100-5 C18 column (250 mm × 4.6 mm, with 5 μm particle size, AKZO NOBEL, Gothenburg, Sweden). The mobile phases used were (A) acetonitrile and (B) 0.01% (v/v) phosphoric acid water solution. The gradient elution was as follows: 5% A from 0 to 5 min, 5 - 18% A from 5 to 20 min, 18% A from 20 to 28.5 min, 18 - 50% A from 28.5 to 50 min, 50 - 55% A from 50 to 57 min, 55% B from 57 to 67 min, 55 - 75% A from 67 to 72 min, 75 - 85% A from 72 to 80 min a d 85 - 5% A from 80 to 90 min at a flow rate of 1.0 mL⋅min^−1^. The signal was monitored at 230 nm. The injection volume of samples was 10 μL.

The professional software "Similarity Evaluation System for Chromatographic Fingerprint of Traditional Chinese Medicine" (Version 2004A, SES software) was used for evaluating the similarities between samples. The reference chromatogram was generated with average data.

Sample no. 1 was selected as a representative sample to validate the method for fingerprint analysis. The method precision and reproducibility were evaluated by the analysis of three injections of the sample solution and four batches of the sample solution prepared independently from sample no. 1. The RSD values of the relative retention time (RRT) and the relative peak area (RPA) for some characteristic peaks are shown. The stability study of the sample was performed within 24 h (0, 4, 8, 12, 24 h). RSDs of RRT and RPA in the precision test (n = 6) were found in the range of 0.01 - 0.06% and 0.38 - 4.60%, respectively. Reproducibility (n = 6) for both RRT (0.01 - 0.06% RSD) and RPA (0.97 - 1.04% RSD) was acceptable. The stability (RSD) was found to be ≤0.05% for RRT and ≤1.27% for RPA, indicating that the sample was stable for 24 h. In total, the RSDs of RTT were less than 0.06% < 2%, and the RSDs of RPA were less than 4.60% < 7%; thus, the adequate precision, reproducibility and stability of the analysis were demonstrated. Supplementary Table S7 summarizes the data.

A fingerprint of Liuweiwuling was then established (Supplementary Figure S3). Supplementary Table S8 shows the similarity values of each sample. The closer the similarity value was to 1, the more similar was the chromatogram to the reference chromatogram. As is shown in Table S8, the similarity values of all 12 samples were higher than 0.979, using the angle cosine method based on common peak by the similarity evaluation system A version of Chinese traditional Chinese medicine chromatographic fingerprint). Table S9 shows the quantitative analysis result of all 12 samples, demonstrating the stability of the sample.

**Table S7** | Injection precision, reproducibility and stability fingerprint analysis

| Peak no. | RSDs of RRT (%) (n=12) | | |  | RSDs of RPA (%) (n=12) | | |
| --- | --- | --- | --- | --- | --- | --- | --- |
|  | Precision | Reproducibility | Stability |  | Precision | Reproducibility | Stability |
| 1 | 0.01 | 0.01 | 0.01 |  | 4.60 | 1.00 | 0.09 |
| 2 | 0.06 | 0.06 | 0.05 |  | 3.85 | 0.97 | 0.25 |
| 3 | 0.01 | 0.01 | 0.01 |  | 0.52 | 0.99 | 0.53 |
| 4 | 0.01 | 0.01 | 0.01 |  | 0.50 | 1.02 | 0.96 |
| 5 | 0.01 | 0.01 | 0.01 |  | 0.38 | 1.01 | 1.27 |
| 6 | 0.01 | 0.01 | 0.01 |  | 0.46 | 0.97 | 0.52 |

**Figure S3** | **Chemical fingerprint of Liuweiwuling nos. 1-12 (S1-S12) were used to construct a fingerprint.** (Peaks were identified by retention time. Peak a for salidroside; peak b for phillyrin; peak c for schisandrol A; peak d for schizandrol A; peak e for schizandrin A and peak f for schizandrin B)

| **Table S8** \| The similarities of chromatograms of each sample | | | |
| --- | --- | --- | --- |
| No. | Similarity | No. | Similarity |
| 1 | 0.979 | 7 | 0.984 |
| 2 | 0.985 | 8 | 0.993 |
| 3 | 0.990 | 9 | 0.992 |
| 4 | 0.987 | 10 | 0.989 |
| 5 | 0.984 | 11 | 0.992 |
| 6 | 0.992 | 12 | 0.981 |

| **Table S9** \| Comparison of area of main peaks in HPLC of 12 batches of Liuweiwuling | | | | | | | |
| --- | --- | --- | --- | --- | --- | --- | --- |
| Batch | peak 1 | peak 2 | peak 3 | peak 4 | peak 5 | peak 6 | Total area of main peaks |
| 1 | 248.64 | 264.61 | 746.32 | 398.49 | 511.86 | 227.77 | 2397.69 |
| 2 | 241.91 | 272.43 | 758.51 | 405.78 | 519.72 | 231.37 | 2429.72 |
| 3 | 245.01 | 263.12 | 746.38 | 416.73 | 511.89 | 228.12 | 2411.25 |
| 4 | 247.17 | 267.87 | 748.63 | 401.32 | 513.88 | 227.19 | 2406.06 |
| 5 | 247.81 | 264.32 | 751.08 | 401.78 | 516.18 | 229.57 | 2410.74 |
| 6 | 254.37 | 381.35 | 619.22 | 507.45 | 543.73 | 362.36 | 2668.48 |
| 7 | 272.33 | 377.99 | 620.69 | 442.03 | 543.99 | 361.61 | 2618.64 |
| 8 | 274.84 | 377.89 | 622.93 | 444.3 | 544.74 | 364.65 | 2629.35 |
| 9 | 272.99 | 384.92 | 623.04 | 444.95 | 541.92 | 364.44 | 2632.26 |
| 10 | 274.63 | 385.04 | 625.69 | 446.68 | 547.39 | 362.51 | 2641.94 |
| 11 | 246.108 | 266.47 | 750.184 | 404.82 | 514.706 | 228.804 | 2411.092 |
| 12 | 269.832 | 381.438 | 622.314 | 457.082 | 544.354 | 363.114 | 2638.134 |
| Note: Total area of main peaks, A_T_=A_i_, A_T_ is the average total area of 12 batches of Liuweiwuling samples | | | | | | | |

- **Prediction of the underlying mechanisms of Liuweiwuling acting on liver failure by network pharmacology**

For the construction of network composed of herb-chemical components and candidate drug targets, either known relationship or predicted relationship of components and drug targets can be used. In this article, drug targets were predicted with Pharmmapper by the molecular docking of herb-chemical components to 2241 known human proteins (<http://59.78.96.61/pharmmapper/>). Furthermore, the protein-protein interactions were also applied to connect this network and liver diseases targets, the unconnected disease targets will be discarded. Since one target may map to different diseases, the new usage of TCM will be provided by disease enrichment analysis of the connected diseases targets. Besides, candidate drug target means the target that the drug component may directly effect on, which was signed as azure circular node or yellow rectangle node in the network according to whether it was the disease target.

**Figure S4** | **Prediction of the underlying mechanisms of Liuweiwuling acting on liver failure by network pharmacology.** (a) The primary network of compound–target–disease of Liuweiwuling; (b) Interaction network of the major candidate drug targets extracted from (a); (c) Interaction network of the composed herb-chemical component-candidate drug target-major liver failure target network from (b); (d) Multi-level network of the main efficacy of each composed herb extracted from (c). Notes: The red triangles represent the active chemical constituents of Liuweiwuling; the azure dots represent the indirect targets for drugs; the yellow dots represent the targets of the specific disease of liver failure; the yellow squares represent the common targets of herbs and liver failure; the purple dots represent the interactional proteins with the targets of liver failure and the drugs and orange hexagon nodes are the six herbs that constitute Liuweiwuling: *Schisandrae Chinensis* Fructus (WWZ), *Ligustri Lucidi* Fructus (NZZ), *Forsythiae* Fructus (LQ), *Sonchus brachyotus* DC. (BJC), *Curcumae* Rhizoma (EZ) and *Ganoderma* Spore (LZ).

- **Major candidate drug targets of Liuweiwuling for treating liver failure**

| **Table S10** \| Major candidate drug targets of Liuweiwuling for treating liver failure | | |
| --- | --- | --- |
| Entry | Entry name | Protein names |
| P03372 | ESR1_HUMAN | Oestrogen receptor (ER) |
| P06239 | LCK_HUMAN | Tyrosine-protein kinase Lck (p56-LCK) |
| P07900 | HS90A_HUMAN | Heat shock protein HSP 90-alpha |
| P10276 | RARA_HUMAN | Retinoic acid receptor alpha (RAR-alpha) |
| P11362 | FGFR1_HUMAN | Fibroblast growth factor receptor 1 (FGFR-1) |
| P12931 | SRC_HUMAN | Proto-oncogene tyrosine-protein kinase Src |
| P19793 | RXRA_HUMAN | Retinoic acid receptor RXR-alpha |
| P22830 | HEMH_HUMAN | Ferrochelatase, mitochondrial (Heme synthase) |
| P28482 | MK01_HUMAN | Mitogen-activated protein kinase 1 (ERK-2) |
| P42574 | CASP3_HUMAN | Caspase-3 (CASP-3) |
| Q02750 | MP2K1_HUMAN | Dual specificity mitogen-activated protein kinase kinase 1 (MAPKK 1) (MKK1) (MEK 1) |
| Q96RI1 | NR1H4_HUMAN | Bile acid receptor (Farnesoid X-activated receptor) |

- **Major liver failure targets associated with the main efficacy of each individual herb in Liuweiwuling**

| **Table S11** \| Major liver failure targets associated with the main efficacy of each individual herb in Liuweiwuling | | | |
| --- | --- | --- | --- |
| Entry | Entry name | Protein names | Associated herbs |
| O15519 | CFLAR_HUMAN | CASP8 and FADD-like apoptosis regulator | WWZ, EZ, LZ, NZZ, LQ |
| P11310 | ACADM_HUMAN | Medium-chain specific acyl-CoA dehydrogenase, mitochondrial | EZ, LZ, WWZ, NZZ |
| P22830 | HEMH_HUMAN | Ferrochelatase, mitochondrial | WWZ, EZ, LZ, NZZ, LQ, BJC |
| P25445 | TNR6_HUMAN | Tumour necrosis factor receptor superfamily member 6 | LZ, NZZ, LQ |
| P53004 | BIEA_HUMAN | Biliverdin reductase A | EZ |
| Q14790 | CASP8_HUMAN | Caspase-8 | WWZ, EZ |
| Q16236 | NF2L2_HUMAN | Nuclear factor erythroid 2-related factor 2 | WWZ, LZ |

For the abbreviation of six drugs in Liuweiwuling, Schisandrae Chinensis Fructus (WWZ), Ligustri Lucidi Fructus (NZZ), Forsythiae Fructus (LQ), Sonchus brachyotus DC. (BJC), Curcumae Rhizoma (EZ) and Ganoderma Spore (LZ).

- **The protein expression and the mRNA levels of major candidate drug targets of Liuweiwuling for treating liver failure**

Hepatic total RNA was isolated by TRIzol reagent (Invitrogen, Thermo Fisher Scientific Inc.). For q RT-PCR, the ﬁrst-strand c DNA was synthesized using RA First Strand cDNA Synthesis Kit (Thermo Fisher Scientific Inc.). q RT-PCR was performed in triplicate with an ABI Prism 7500 sequence detection system and Power SYBR Green PCR Master Mix reagents (Life technologies, Thermo Fisher Scientific Inc.). The speciﬁc primers for RXRα, Hsp90ab1, Hsp90aa1, HEMH, Src, MEK1, Nr1h4, CASP-3, RARα, FGFR1, Lck, ESR1 and GAPDH were purchased from Life technologies (Life technologies, Thermo Fisher Scientific Inc.). Data analysis was performed using the 2−ΔΔCT method for relative quantification. GAPDH expression was used to normalize the obtained data. Primer sequences for mice RXRα, Hsp90ab1, Hsp90aa1, HEMH, Src, MEK1, Nr1h4, CASP-3, RARα, FGFR1, Lck, ESR1 and GAPDH primers were shown as follows.

| **Table S12 \|** Primer sequences used in this manuscript | | |
| --- | --- | --- |
| Target gene | the primer sequences (5'-3') | fragment length (bp) |
| M-GAPDH-S | AGGAGCGAGACCCCACTAACA | 247 |
| M-GAPDH-A | AGGGGGGCTAAGCAGTTGGT | 247 |
| M-lck-S | CGATCTAGTCCGCCATTACACC | 176 |
| M-lck-A | CCATCCACACTTCCCCGAAC | 176 |
| M-ESR1-S | TCTGCCAAGGAGACTCGCTAC | 171 |
| M-ESR1-A | CCTCCGGTTCTTGTCAATGGT | 171 |
| M-ERK-2-S | GTGACCTCAAGCCTTCCAACCT | 208 |
| M-ERK-2-A | ATGCAGCCCACAGACCAAATAT | 208 |
| M-Hsp90ab1-S | ATGATCGGGCAGTTTGGTGT | 234 |
| M-Hsp90ab1-A | CACCACTTCCTTGACCCTCC | 234 |
| M-Hsp90aa1-S | GTTCATTCAGCCACGATGCC | 290 |
| M-Hsp90aa1-A | ACAATGGTCAGGGTTCGGTC | 290 |
| M-HEMH-S | TTCATCGCCAAACGCCGAAC | 174 |
| M-HEMH-A | ATGGACGTACCGGAATCCAA | 174 |
| M-RARα-S | CAGAGCAGCAGTTCCGAAGAGAT | 169 |
| M-RARα-A | TGTTCTTCTGGATGCTTCGTCG | 169 |
| M-Fgfr1-S | GGAGGCTACAAGGTTCGCTAT | 103 |
| M-Fgfr1-A | TCCCATACTCATTCTCCACGAT | 103 |
| M-Src-S | ATCACTAGACGGGAATCAGAGCG | 208 |
| M-Src-A | TGTTGAACTGGGTGCGGGAG | 208 |
| M-RXRα-S | CCTCCTTCACCAAGCACATCT | 283 |
| M-RXRα-A | GACTCCACCTCGTTCTCATTCC | 283 |
| M-CASP-3-S | GTCTGACTGGAAAGCCGAAAC | 205 |
| M-CASP-3-A | GACTGGATGAACCACGACCC | 205 |
| M-MEK1-S | TTGGAGCCGACTGCAAATACA | 115 |
| M-MEK1-A | TCCTGGCACTGGGCATTGTA | 115 |
| M-Nr1h4-S | GCACTCCCATTTACAGGCTACG | 187 |
| M-Nr1h4-A | GCCCAGGTTGGAATAGTAAGACG | 187 |

**Figure S5 | The mRNA levels of major candidate drug targets of Liuweiwuling for treating liver failure.** (a-l) The mRNA levels of RXRα, Hsp90ab1, Hsp90aa1, HEMH, Src, MEK1, Nr1h4, CASP-3, RARα, FGFR1, Lck, ESR1 in livers of mice 6 h after GalN/LPS injection. n = 6-8 / group. ‘*’, ‘**’ and ‘***’, P < 0.05, P < 0.01 and P < 0.001, respectively, comparison with the control group. ‘#’, ‘##’ and ‘###’, P < 0.05, P < 0.01 and P < 0.001, respectively, comparison with the model group. All experiments were performed 3 times, and the results are expressed as the mean ± S.D. For the abbreviation of groups in experimental design, control group (Con), model group (Mod), and 16 mg/g Liuweiwuling group (LW4).

**Figure S6 | The protein expression levels of major candidate drug targets of Liuweiwuling for treating liver failure.** For the abbreviation of groups in experimental design, control group (Con), model group (Mod), and 16 mg/g Liuweiwuling group (LW4).

- **Serum aminotransferase levels (alanine aminotransferase [ALT] and aspartate aminotransferase [AST]) following GalN/LPS or TAA injection**

**Figure S7 | Serum aminotransferase levels (alanine aminotransferase [ALT] and aspartate aminotransferase [AST]) following GalN/LPS or TAA injection.** (a) Serum aminotransferase levels (alanine aminotransferase [ALT] and aspartate aminotransferase [AST]) are shown 6 h after treatment with GalN/LPS or the vehicle control. (b) Serum aminotransferase levels (alanine aminotransferase [ALT] and aspartate aminotransferase [AST]) are shown 12 h after the last treatment with TAA or the vehicle control. (n = 6-8 / group, data are the mean ± S.D. ‘*’, ‘**’ and ‘***’ indicate P < 0.05, P < 0.01 and P < 0.001, respectively, versus the control group. ‘#’, ‘##’ and ‘###’ indicate P < 0.05, P < 0.01 and P < 0.001, respectively, versus the model group). For the abbreviation of groups in experimental design, control group (Con), model group (Mod), positive drug group (Bic), 0.5 mg/g Liuweiwuling group (LW1), 2 mg/g Liuweiwuling group (LW2), 8 mg/g Liuweiwuling group (LW3), and 16 mg/g Liuweiwuling group (LW4).

- **A TUNEL assay 6 h after GalN/LPS treatment or 12 h after the last treatment with TAA for the different groups**

**Figure S8 | A TUNEL assay 6 h after GalN/LPS treatment or 12 h after the last treatment with TAA for the different groups following GalN/LPS or TAA injection.** (a) A TUNEL assay is shown 6 h after treatment with GalN/LPS or the vehicle control. (b) A TUNEL assay is shown 12 h after the last treatment with TAA or the vehicle control. (n = 6-8 / group, data are the mean ± S.D. ‘*’, ‘**’ and ‘***’ indicate P < 0.05, P < 0.01 and P < 0.001, respectively, versus the control group. ‘#’, ‘##’ and ‘###’ indicate P < 0.05, P < 0.01 and P < 0.001, respectively, versus the model group). For the abbreviation of groups in experimental design, control group (Con), model group (Mod), positive drug group (Bic), 0.5 mg/g Liuweiwuling group (LW1), 2 mg/g Liuweiwuling group (LW2), 8 mg/g Liuweiwuling group (LW3), and 16 mg/g Liuweiwuling group (LW4).

- **The greyscale of western blot results in the apoptosis pathways**

The expression of target proteins was calculated by grey scanning for western blot analyses, and the results are shown in Supplementary Figure S9.

**Figure S9** | **Western blot analyses of target proteins in apoptosis pathways after hepatotoxin injection.** (a-i) Western blot analyses of target proteins in the apoptosis pathways 6 h after GalN/LPS injection. (j-o) Western blot analyses of target proteins in the apoptosis pathways 12 h after TAA consecutive injection. ‘*’, ‘**’ and ‘***’, P < 0.05, P < 0.01 and P < 0.001, respectively, comparison with the control group. ‘#’, ‘##’ and ‘###’, P < 0.05, P < 0.01 and P < 0.001, respectively, comparison with the model group. All experiments were performed 3 times, and the results are expressed as the mean ± S.D. For the abbreviation of groups in experimental design, control group (Con), model group (Mod), positive drug group (Bic), 0.5 mg/g Liuweiwuling group (LW1), 2 mg/g Liuweiwuling group (LW2), 8 mg/g Liuweiwuling group (LW3), and 16 mg/g Liuweiwuling group (LW4).

- **Liuweiwuling administration suppresses TNF-α, FADD and Cyt C protein expression in the hepatocytes of GalN/LPS-treated mice by immunohistochemistry**

**Figure S10** | **Liuweiwuling administration suppresses TNF-α, FADD and Cyt C protein expression in the hepatocytes of GalN/LPS-treated mice by immunohistochemistry (a-c).** The data are expressed as the mean ± S.D. ‘*’, ‘**’ and ‘***’, P < 0.05, P < 0.01 and P < 0.001, respectively, in comparison with the control group. ‘#’, ‘##’ and ‘###’ indicate P < 0.05, P < 0.01 and P < 0.001, respectively, in comparison with the model group (n = 6-8 / group). For the abbreviation of groups in experimental design, control group (Con), model group (Mod), positive drug group (Bic), 0.5 mg/g Liuweiwuling group (LW1), 2 mg/g Liuweiwuling group (LW2), 8 mg/g Liuweiwuling group (LW3), and 16 mg/g Liuweiwuling group (LW4).

- **Liuweiwuling and its decomposed ingredients protect against GalN/LPS-induced ALF**

**Figure S11 | Liuweiwuling and its decomposed ingredients protect against GalN/LPS-induced ALF.** (a) Serum transaminase levels (alanine aminotransferase [ALT] and aspartate aminotransferase [AST]) are shown 6 h after treatment with GalN/LPS or the vehicle control. (b) A TUNEL assay 6 h after the GalN/LPS treatment of the different groups as indicated. (n = 6-8 / group, data are the mean ± S.D. ‘*’, ‘**’ and ‘***’ indicate P < 0.05, P < 0.01 and P < 0.001, respectively, versus the control group. ‘#’, ‘##’ and ‘###’ indicate P < 0.05, P < 0.01 and P < 0.001, respectively, versus the model group). For the abbreviation of groups in experimental design, control group (Con), model group (Mod), positive drug group (Bic), 16 mg/g Liuweiwuling group (LW4), JG (the mixture of four herbs, *Chinensis* Fructus, *Ligustri Lucidi* Fructus, *Forsythiae* Fructus and *Sonchus brachyotus* DC) group (JG), *Ganoderma* Spore group (LZ), and *Curcumae* Rhizoma group (EZ).

- **The protective effect of Liuweiwuling and its separate ingredients on serum TNF-α in GalN/LPS-injected mice**

The serum TNF-α content was measured with commercial ELISA kits (purchased from the Dakewe for Biological Technology Co., Ltd., Beijing, China) according to the manufacturer’s protocol. Liuweiwuling is composed of *Schisandrae Chinensis* Fructus (WWZ), *Ligustri Lucidi* Fructus (NZZ), *Forsythiae* Fructus (LQ), *Sonchus brachyotus* DC. (BJC), *Curcumae* Rhizoma (EZ) and *Ganoderma* Spore (LZ). We combined WWZ, NZZ, LQ and BJC as a mixture (JG) based on a network pharmacology analysis. GalN/LPS caused increased levels of TNF-α when compared with control mice, which was decreased by Liuweiwuling and JG as compared with other groups. The results demonstrated the synergistic efficacy of Liuweiwuling on liver failure.

**Figure S12** | **The protective effect of Liuweiwuling and its separate ingredients (JG, LZ, EZ) on serum TNF-α in GalN/LPS-injected mice by** **ELISA.** Mice were pretreated with Liuweiwuling and its individual ingredients as described in Fig. 6a. The serum TNF-α levels were determined by ELISA. The data are expressed as the mean ± S.D. ‘*’, ‘**’ and ‘***’ and indicate P < 0.05, P < 0.01 and P < 0.001, respectively, in comparison with the control group. ‘#’, ‘##’ and ‘###’ indicate P < 0.05, P < 0.01 and P < 0.001, respectively, in comparison with the model group (n = 6-8 / group). For the abbreviation of groups in experimental design, control group (Con), model group (Mod), positive drug group (Bic), 16 mg/g Liuweiwuling group (LW4), JG (the mixture of four herbs, *Chinensis* Fructus, *Ligustri Lucidi* Fructus, *Forsythiae* Fructus and *Sonchus brachyotus* DC) group (JG), *Ganoderma* Spore group (LZ), and *Curcumae* Rhizoma group (EZ).

- **Macroscopic appearance and H&E staining of all liver samples 6 h after GalN/LPS or 12 h after TAA treatment of respective groups in the experiment**

Macroscopic appearance and H&E staining of all the livers of GalN/LPS-injected or TAA-injected mice in the experiment were shown as figures below.

**Figure S13 | Macroscopic appearance and H&E staining of all liver samples 6 h after GalN/LPS treatment of control (Con) groups as indicated.**

**Figure S14 | Macroscopic appearance and H&E staining of all liver samples 6 h after GalN/LPS treatment of model (Mod) groups as indicated.**

**Figure S15 | Macroscopic appearance and H&E staining of all liver samples 6 h after GalN/LPS treatment of bicyclol (Bic) groups as indicated.**

**Figure S16 | Macroscopic appearance and H&E staining of all liver samples 6 h after GalN/LPS treatment of LW1 groups as indicated.**

**Figure S17 | Macroscopic appearance and H&E staining of all liver samples 6 h after GalN/LPS treatment of LW2 groups as indicated.**

**Figure S18 | Macroscopic appearance and H&E staining of all liver samples 6 h after GalN/LPS treatment of LW3 groups as indicated.**

**Figure S19 | Macroscopic appearance and H&E staining of all liver samples 6 h after GalN/LPS treatment of LW4 groups as indicated.**

**Figure S20 | H&E staining of all liver samples 12 h after the last treatment with TAA of Con groups as indicated.**

**Figure S21 | H&E staining of all liver samples 12 h after the last treatment with TAA of Mod groups as indicated.**

**Figure S22 | H&E staining of all liver samples 12 h after the last treatment with TAA of Bic groups as indicated.**

**Figure S23 | H&E staining of all liver samples 12 h after the last treatment with TAA of LW1 groups as indicated.**

**Figure S24 | H&E staining of all liver samples 12 h after the last treatment with TAA of LW2 groups as indicated.**

**Figure S25 | H&E staining of all liver samples 12 h after the last treatment with TAA of LW3 groups as indicated.**

**Figure S26 | H&E staining of all liver samples 12 h after the last treatment with TAA of LW4 groups as indicated.**

- **The efficacy of Liuweiwuling on promoting cell proliferation for treating liver failure by immunohistochemistry**

**Figure S27 | The efficacy of Liuweiwuling on promoting cell proliferation for treating liver failure by** **immunohistochemistry.** (a) An immunohistochemical analysis of PCNA is shown 6 h after treatment with GalN/LPS or the vehicle control. (b) An immunohistochemical analysis of PCNA is shown 12 h after the last treatment with TAA or the vehicle control. (n = 6-8 / group, data are the mean ± SEM, ‘*’, ‘**’ and ‘***’ indicate P < 0.05, P < 0.01 and P < 0.001, respectively, versus the control group. ‘#’, ‘##’ and ‘###’ indicate P < 0.05, P < 0.01 and P < 0.001, respectively, versus the model group). For the abbreviation of groups in experimental design, control group (Con), model group (Mod), and 16 mg/g Liuweiwuling group (LW4).

- **The compliance of the grouping of gels/blots cropped from different parts of the same gel, or from different gels, fields in the main manuscript**

**Figure S28** | **The compliance of the grouping of gels/blots cropped from different parts of the same gel, or from different gels, fields.** (a-i) The compliance of the grouping of gels/blots for TNFR, Fas, FasL, Bcl-2, Bcl-xL, CASP-8, CASP-3 (35 kDa), CASP-3 (17 kDa) and GAPDH in the apoptosis pathways 6 h after GalN/LPS injection. (j-o) The compliance of the grouping of gels/blots for CASP-8, CASP-3 (35 kDa), CASP-3 (17 kDa), ACTIN, CASP-9 and Bid in the apoptosis pathways 12 h after TAA consecutive injection.

- **All drug targets and diseases targets associated with Liuweiwuling or liver failure**

**Table S13 |** All drug targets associated with Liuweiwuling

| No. | Entry | Entry name | Protein names |
| --- | --- | --- | --- |
| 1 | O00204 | ST2B1_HUMAN | Sulfotransferase family cytosolic 2B member 1 |
| 2 | O14717 | TRDMT_HUMAN | tRNA (cytosine(38)-C(5))-methyltransferase |
| 3 | O14727 | APAF_HUMAN | Apoptotic protease-activating factor 1 |
| 4 | O14757 | CHK1_HUMAN | Serine/threonine-protein kinase Chk1 |
| 5 | O14965 | AURKA_HUMAN | Aurora kinase A |
| 6 | O15382 | BCAT2_HUMAN | Branched-chain-amino-acid aminotransferase, mitochondrial |
| 7 | O15530 | PDPK1_HUMAN | 3-phosphoinositide-dependent protein kinase 1 |
| 8 | O15540 | FABP7_HUMAN | Fatty acid-binding protein, brain |
| 9 | O43252 | PAPS1_HUMAN | Bifunctional 3'-phosphoadenosine 5'-phosphosulfate synthase 1 |
| 10 | O43617 | TPPC3_HUMAN | Trafficking protein particle complex subunit 3 |
| 11 | O60674 | JAK2_HUMAN | Tyrosine-protein kinase JAK2 |
| 12 | O60760 | HPGDS_HUMAN | Hematopoietic prostaglandin D synthase |
| 13 | O75469 | NR1I2_HUMAN | Nuclear receptor subfamily 1 group I member 2 |
| 14 | O76054 | S14L2_HUMAN | SEC14-like protein 2 |
| 15 | O76074 | PDE5A_HUMAN | cGMP-specific 3',5'-cyclic phosphodiesterase |
| 16 | P00374 | DYR_HUMAN | Dihydrofolate reductase |
| 17 | P00492 | HPRT_HUMAN | Hypoxanthine-guanine phosphoribosyltransferase |
| 18 | P00519 | ABL1_HUMAN | Tyrosine-protein kinase ABL1 |
| 19 | P00533 | EGFR_HUMAN | Epidermal growth factor receptor |
| 20 | P00734 | THRB_HUMAN | Prothrombin |
| 21 | P00742 | FA10_HUMAN | Coagulation factor X |
| 22 | P00797 | RENI_HUMAN | Renin |
| 23 | P00918 | CAH2_HUMAN | Carbonic anhydrase 2 |
| 24 | P01009 | A1AT_HUMAN | Alpha-1-antitrypsin |
| 25 | P01112 | RASH_HUMAN | GTPase Hras |
| 26 | P05019 | IGF1_HUMAN | Insulin-like growth factor I |
| 27 | P02679 | FIBG_HUMAN | Fibrinogen gamma chain |
| 28 | P02753 | RET4_HUMAN | Retinol-binding protein 4 |
| 29 | P02766 | TTHY_HUMAN | Transthyretin |
| 30 | P02768 | ALBU_HUMAN | Serum albumin |
| 31 | P02774 | VTDB_HUMAN | Vitamin D-binding protein |
| 32 | P03372 | ESR1_HUMAN | Estrogen receptor |
| 33 | P04035 | HMDH_HUMAN | 3-hydroxy-3-methylglutaryl-coenzyme A reductase |
| 34 | P04062 | GLCM_HUMAN | Glucosylceramidase |
| 35 | P04150 | GCR_HUMAN | Glucocorticoid receptor |
| 36 | P04181 | OAT_HUMAN | Ornithine aminotransferase, mitochondrial |
| 37 | P04278 | SHBG_HUMAN | Sex hormone-binding globulin |
| 38 | P04746 | AMYP_HUMAN | Pancreatic alpha-amylase |
| 39 | P04818 | TYSY_HUMAN | Thymidylate synthase |
| 40 | P05413 | FABPH_HUMAN | Fatty acid-binding protein, heart |
| 41 | P06126 | CD1A_HUMAN | T-cell surface glycoprotein CD1a |
| 42 | P06213 | INSR_HUMAN | Insulin receptor |
| 43 | P06239 | LCK_HUMAN | Tyrosine-protein kinase Lck |
| 44 | P06401 | PRGR_HUMAN | Progesterone receptor |
| 45 | P06702 | S10A9_HUMAN | Protein S100-A9 |
| 46 | P06730 | IF4E_HUMAN | Eukaryotic translation initiation factor 4E |
| 47 | P06737 | PYGL_HUMAN | Glycogen phosphorylase, liver form |
| 48 | P06744 | G6PI_HUMAN | Glucose-6-phosphate isomerase |
| 49 | P07359 | GP1BA_HUMAN | Platelet glycoprotein Ib alpha chain |
| 50 | P07686 | HEXB_HUMAN | Beta-hexosaminidase subunit beta |
| 51 | P07858 | CATB_HUMAN | Cathepsin B |
| 52 | P07900 | HS90A_HUMAN | Heat shock protein HSP 90-alpha |
| 53 | P08069 | IGF1R_HUMAN | Insulin-like growth factor 1 receptor |
| 54 | P08235 | MCR_HUMAN | Mineralocorticoid receptor |
| 55 | P08238 | HS90B_HUMAN | Heat shock protein HSP 90-beta |
| 56 | P08246 | ELNE_HUMAN | Neutrophil elastase |
| 57 | P08253 | MMP2_HUMAN | 72 kDa type IV collagenase |
| 58 | P08254 | MMP3_HUMAN | Stromelysin-1 |
| 59 | P08263 | GSTA1_HUMAN | Glutathione S-transferase A1 |
| 60 | P08473 | NEP_HUMAN | Neprilysin |
| 61 | P08581 | MET_HUMAN | Hepatocyte growth factor receptor |
| 62 | P08631 | HCK_HUMAN | Tyrosine-protein kinase HCK |
| 63 | P09211 | GSTP1_HUMAN | Glutathione S-transferase P |
| 64 | P09874 | PARP1_HUMAN | Poly [ADP-ribose] polymerase 1 |
| 65 | P09960 | LKHA4_HUMAN | Leukotriene A-4 hydrolase |
| 66 | P0CG30 | GSTT2_HUMAN | Glutathione S-transferase theta-2B |
| 67 | P10114 | RAP2A_HUMAN | Ras-related protein Rap-2a |
| 68 | P10275 | ANDR_HUMAN | Androgen receptor |
| 69 | P10276 | RARA_HUMAN | Retinoic acid receptor alpha |
| 70 | P10632 | CP2C8_HUMAN | Cytochrome P450 2C8 |
| 71 | P10721 | KIT_HUMAN | Mast/stem cell growth factor receptor Kit |
| 72 | P10826 | RARB_HUMAN | Retinoic acid receptor beta |
| 73 | P10827 | THA_HUMAN | Thyroid hormone receptor alpha |
| 74 | P10828 | THB_HUMAN | Thyroid hormone receptor beta |
| 75 | P11086 | PNMT_HUMAN | Phenylethanolamine N-methyltransferase |
| 76 | P11309 | PIM1_HUMAN | Serine/threonine-protein kinase pim-1 |
| 77 | P11362 | FGFR1_HUMAN | Fibroblast growth factor receptor 1 |
| 78 | P11473 | VDR_HUMAN | Vitamin D3 receptor |
| 79 | P11586 | C1TC_HUMAN | C-1-tetrahydrofolate synthase, cytoplasmic |
| 80 | P11712 | CP2C9_HUMAN | Cytochrome P450 2C9 |
| 81 | P11766 | ADHX_HUMAN | Alcohol dehydrogenase class-3 |
| 82 | P12724 | ECP_HUMAN | Eosinophil cationic protein |
| 83 | P12821 | ACE_HUMAN | Angiotensin-converting enzyme |
| 84 | P12931 | SRC_HUMAN | Proto-oncogene tyrosine-protein kinase Src |
| 85 | P13631 | RARG_HUMAN | Retinoic acid receptor gamma |
| 86 | P14061 | DHB1_HUMAN | Estradiol 17-beta-dehydrogenase 1 |
| 87 | P14324 | FPPS_HUMAN | Farnesyl pyrophosphate synthase |
| 88 | P14555 | PA2GA_HUMAN | Phospholipase A2, membrane associated |
| 89 | P14780 | MMP9_HUMAN | Matrix metalloproteinase-9 |
| 90 | P15056 | BRAF_HUMAN | Serine/threonine-protein kinase B-raf |
| 91 | P15121 | ALDR_HUMAN | Aldose reductase |
| 92 | P15559 | NQO1_HUMAN | NAD(P)H dehydrogenase [quinone] 1 |
| 93 | P16152 | CBR1_HUMAN | Carbonyl reductase [NADPH] 1 |
| 94 | P16442 | BGAT_HUMAN | Histo-blood group ABO system transferase |
| 95 | P16444 | DPEP1_HUMAN | Dipeptidase 1 |
| 96 | P17612 | KAPCA_HUMAN | cAMP-dependent protein kinase catalytic subunit alpha |
| 97 | P17752 | TPH1_HUMAN | Tryptophan 5-hydroxylase 1 |
| 98 | P17931 | LEG3_HUMAN | Galectin-3 |
| 99 | P18031 | PTN1_HUMAN | Tyrosine-protein phosphatase non-receptor type 1 |
| 100 | P19367 | HXK1_HUMAN | Hexokinase-1 |
| 101 | P19793 | RXRA_HUMAN | Retinoic acid receptor RXR-alpha |
| 102 | P20248 | CCNA2_HUMAN | Cyclin-A2 |
| 103 | P20339 | RAB5A_HUMAN | Ras-related protein Rab-5A |
| 104 | P20701 | ITAL_HUMAN | Integrin alpha-L |
| 105 | P21397 | AOFA_HUMAN | Amine oxidase [flavin-containing] A |
| 106 | P22102 | PUR2_HUMAN | Trifunctional purine biosynthetic protein adenosine-3 |
| 107 | P22830 | HEMH_HUMAN | Ferrochelatase, mitochondrial |
| 108 | P23381 | SYWC_HUMAN | Tryptophan--tRNA ligase, cytoplasmic |
| 109 | P23677 | IP3KA_HUMAN | Inositol-trisphosphate 3-kinase A |
| 110 | P23919 | KTHY_HUMAN | Thymidylate kinase |
| 111 | P23946 | CMA1_HUMAN | Chymase |
| 112 | P24941 | CDK2_HUMAN | Cyclin-dependent kinase 2 |
| 113 | P25774 | CATS_HUMAN | Cathepsin S |
| 114 | P27338 | AOFB_HUMAN | Amine oxidase [flavin-containing] B |
| 115 | P27487 | DPP4_HUMAN | Dipeptidyl peptidase 4 |
| 116 | P27707 | DCK_HUMAN | Deoxycytidine kinase |
| 117 | P27986 | P85A_HUMAN | Phosphatidylinositol 3-kinase regulatory subunit alpha |
| 118 | P28161 | GSTM2_HUMAN | Glutathione S-transferase Mu 2 |
| 119 | P28482 | MK01_HUMAN | Mitogen-activated protein kinase 1 |
| 120 | P28702 | RXRB_HUMAN | Retinoic acid receptor RXR-beta |
| 121 | P28845 | DHI1_HUMAN | Corticosteroid 11-beta-dehydrogenase isozyme 1 |
| 122 | P29218 | IMPA1_HUMAN | Inositol monophosphatase 1 |
| 123 | P29373 | RABP2_HUMAN | Cellular retinoic acid-binding protein 2 |
| 124 | P29466 | CASP1_HUMAN | Caspase-1 |
| 125 | P30043 | BLVRB_HUMAN | Flavin reductase |
| 126 | P30613 | KPYR_HUMAN | Pyruvate kinase PKLR |
| 127 | P31749 | AKT1_HUMAN | RAC-alpha serine/threonine-protein kinase |
| 128 | P34896 | GLYC_HUMAN | Serine hydroxymethyltransferase, cytosolic |
| 129 | P35221 | CTNA1_HUMAN | Catenin alpha-1 |
| 130 | P35398 | RORA_HUMAN | Nuclear receptor ROR-alpha |
| 131 | P35557 | HXK4_HUMAN | Glucokinase |
| 132 | P35558 | PCKGC_HUMAN | Phosphoenolpyruvate carboxykinase, cytosolic |
| 133 | P35968 | VGFR2_HUMAN | Vascular endothelial growth factor receptor 2 |
| 134 | P36873 | PP1G_HUMAN | Serine/threonine-protein phosphatase PP1-gamma catalytic subunit |
| 135 | P36897 | TGFR1_HUMAN | TGF-beta receptor type-1 |
| 136 | P37231 | PPARG_HUMAN | Peroxisome proliferator-activated receptor gamma |
| 137 | P39900 | MMP12_HUMAN | Macrophage metalloelastase |
| 138 | P42330 | AK1C3_HUMAN | Aldo-keto reductase family 1 member C3 |
| 139 | P42574 | CASP3_HUMAN | Caspase-3 |
| 140 | P42768 | WASP_HUMAN | Wiskott-Aldrich syndrome protein |
| 141 | P43235 | CATK_HUMAN | Cathepsin K |
| 142 | P43403 | ZAP70_HUMAN | Tyrosine-protein kinase ZAP-70 |
| 143 | P43405 | KSYK_HUMAN | Tyrosine-protein kinase SYK |
| 144 | P45452 | MMP13_HUMAN | Collagenase 3 |
| 145 | P48449 | ERG7_HUMAN | Lanosterol synthase |
| 146 | P49137 | MAPK2_HUMAN | MAP kinase-activated protein kinase 2 |
| 147 | P49354 | FNTA_HUMAN | Protein farnesyltransferase/geranylgeranyltransferase type-1 subunit alpha |
| 148 | P49638 | TTPA_HUMAN | Alpha-tocopherol transfer protein |
| 149 | P49773 | HINT1_HUMAN | Histidine triad nucleotide-binding protein 1 |
| 150 | P49841 | GSK3B_HUMAN | Glycogen synthase kinase-3 beta |
| 151 | P49888 | ST1E1_HUMAN | Estrogen sulfotransferase |
| 152 | P50135 | HNMT_HUMAN | Histamine N-methyltransferase |
| 153 | P50225 | ST1A1_HUMAN | Sulfotransferase 1A1 |
| 154 | P50579 | MAP2_HUMAN | Methionine aminopeptidase 2 |
| 155 | P51161 | FABP6_HUMAN | Gastrotropin |
| 156 | P52333 | JAK3_HUMAN | Tyrosine-protein kinase JAK3 |
| 157 | P52732 | KIF11_HUMAN | Kinesin-like protein KIF11 |
| 158 | P52895 | AK1C2_HUMAN | Aldo-keto reductase family 1 member C2 |
| 159 | P53355 | DAPK1_HUMAN | Death-associated protein kinase 1 |
| 160 | P53779 | MK10_HUMAN | Mitogen-activated protein kinase 10 |
| 161 | P54760 | EPHB4_HUMAN | Ephrin type-B receptor 4 |
| 162 | P55055 | NR1H2_HUMAN | Oxysterols receptor LXR-beta |
| 163 | P55263 | ADK_HUMAN | Adenosine kinase |
| 164 | P56817 | BACE1_HUMAN | Beta-secretase 1 |
| 165 | P60568 | IL2_HUMAN | Interleukin-2 |
| 166 | P60953 | CDC42_HUMAN | Cell division control protein 42 homolog |
| 167 | P61812 | TGFB2_HUMAN | Transforming growth factor beta-2 |
| 168 | P62158 | CALM_HUMAN | Calmodulin |
| 169 | P62508 | ERR3_HUMAN | Estrogen-related receptor gamma |
| 170 | P62942 | FKB1A_HUMAN | Peptidyl-prolyl cis-trans isomerase FKBP1A |
| 171 | P62993 | GRB2_HUMAN | Growth factor receptor-bound protein 2 |
| 172 | P78536 | ADA17_HUMAN | Disintegrin and metalloproteinase domain-containing protein 17 |
| 173 | P80188 | NGAL_HUMAN | Neutrophil gelatinase-associated lipocalin |
| 174 | Q00534 | CDK6_HUMAN | Cyclin-dependent kinase 6 |
| 175 | Q00688 | FKBP3_HUMAN | Peptidyl-prolyl cis-trans isomerase FKBP3 |
| 176 | Q00796 | DHSO_HUMAN | Sorbitol dehydrogenase |
| 177 | Q00987 | MDM2_HUMAN | E3 ubiquitin-protein ligase Mdm2 |
| 178 | Q02127 | PYRD_HUMAN | Dihydroorotate dehydrogenase (quinone), mitochondrial |
| 179 | Q02750 | MP2K1_HUMAN | Dual specificity mitogen-activated protein kinase kinase 1 |
| 180 | Q02763 | TIE2_HUMAN | Angiopoietin-1 receptor |
| 181 | Q03181 | PPARD_HUMAN | Peroxisome proliferator-activated receptor delta |
| 182 | Q03518 | TAP1_HUMAN | Antigen peptide transporter 1 |
| 183 | Q04759 | KPCT_HUMAN | Protein kinase C theta type |
| 184 | Q04760 | LGUL_HUMAN | Lactoylglutathione lyase |
| 185 | Q04828 | AK1C1_HUMAN | Aldo-keto reductase family 1 member C1 |
| 186 | Q06124 | PTN11_HUMAN | Tyrosine-protein phosphatase non-receptor type 11 |
| 187 | Q06187 | BTK_HUMAN | Tyrosine-protein kinase BTK |
| 188 | Q06520 | ST2A1_HUMAN | Bile salt sulfotransferase |
| 189 | Q07343 | PDE4B_HUMAN | cAMP-specific 3',5'-cyclic phosphodiesterase 4B |
| 190 | Q07817 | B2CL1_HUMAN | Bcl-2-like protein 1 |
| 191 | Q07869 | PPARA_HUMAN | Peroxisome proliferator-activated receptor alpha |
| 192 | Q08188 | TGM3_HUMAN | Protein-glutamine gamma-glutamyltransferase E |
| 193 | Q08499 | PDE4D_HUMAN | cAMP-specific 3',5'-cyclic phosphodiesterase 4D |
| 194 | Q08881 | ITK_HUMAN | Tyrosine-protein kinase ITK/TSK |
| 195 | Q10588 | BST1_HUMAN | ADP-ribosyl cyclase/cyclic ADP-ribose hydrolase 2 |
| 196 | Q13126 | MTAP_HUMAN | S-methyl-5'-thioadenosine phosphorylase |
| 197 | Q13133 | NR1H3_HUMAN | Oxysterols receptor LXR-alpha |
| 198 | Q13231 | CHIT1_HUMAN | Chitotriosidase-1 |
| 199 | Q14376 | GALE_HUMAN | UDP-glucose 4-epimerase |
| 200 | Q14541 | HNF4G_HUMAN | Hepatocyte nuclear factor 4-gamma |
| 201 | Q14994 | NR1I3_HUMAN | Nuclear receptor subfamily 1 group I member 3 |
| 202 | Q15075 | EEA1_HUMAN | Early endosome antigen 1 |
| 203 | Q15119 | PDK2_HUMAN | [Pyruvate dehydrogenase] kinase isozyme 2, mitochondrial |
| 204 | Q15303 | ERBB4_HUMAN | Receptor tyrosine-protein kinase erbB-4 |
| 205 | Q16222 | UAP1_HUMAN | UDP-N-acetylhexosamine pyrophosphorylase |
| 206 | Q16539 | MK14_HUMAN | Mitogen-activated protein kinase 14 |
| 207 | Q16772 | GSTA3_HUMAN | Glutathione S-transferase A3 |
| 208 | Q16836 | HCDH_HUMAN | Hydroxyacyl-coenzyme A dehydrogenase, mitochondrial |
| 209 | Q8IV48 | ERI1_HUMAN | 3'-5' exoribonuclease 1 |
| 210 | Q8TEK3 | DOT1L_HUMAN | Histone-lysine N-methyltransferase, H3 lysine-79 specific |
| 211 | Q92731 | ESR2_HUMAN | Estrogen receptor beta |
| 212 | Q92831 | KAT2B_HUMAN | Histone acetyltransferase KAT2B |
| 213 | Q93088 | BHMT1_HUMAN | Betaine--homocysteine S-methyltransferase 1 |
| 214 | Q96CA5 | BIRC7_HUMAN | Baculoviral IAP repeat-containing protein 7 |
| 215 | Q96KC2 | ARL5B_HUMAN | ADP-ribosylation factor-like protein 5B |
| 216 | Q96RI1 | NR1H4_HUMAN | Bile acid receptor |
| 217 | Q9BY41 | HDAC8_HUMAN | Histone deacetylase 8 |
| 218 | Q9BZ11 | ADA33_HUMAN | Disintegrin and metalloproteinase domain-containing protein 33 |
| 219 | Q9BZX2 | UCK2_HUMAN | Uridine-cytidine kinase 2 |
| 220 | Q9HAN9 | NMNA1_HUMAN | Nicotinamide/nicotinic acid mononucleotide adenylyltransferase 1 |
| 221 | Q9NPB1 | NT5M_HUMAN | 5'(3')-deoxyribonucleotidase, mitochondrial |
| 222 | Q9NXA8 | SIR5_HUMAN | NAD-dependent protein deacylase sirtuin-5, mitochondrial |
| 223 | P16442 | BGAT_HUMAN | Histo-blood group ABO system transferase |
| 224 | Q9P2T1 | GMPR2_HUMAN | GMP reductase 2 |
| 225 | Q9P2W7 | B3GA1_HUMAN | Galactosylgalactosylxylosylprotein 3-beta-glucuronosyltransferase 1 |
| 226 | Q9UKL6 | PPCT_HUMAN | Phosphatidylcholine transfer protein |
| 227 | Q9UKM7 | MA1B1_HUMAN | Endoplasmic reticulum mannosyl-oligosaccharide 1,2-alpha-mannosidase |
| 228 | Q9UNN8 | EPCR_HUMAN | Endothelial protein C receptor |
| 229 | Q9Y689 | ARL5A_HUMAN | ADP-ribosylation factor-like protein 5A |

**Table S14 |** All diseases targets associated with liver failure

| No. | Entry | Entry name | Protein names |
| --- | --- | --- | --- |
| 1 | P11310 | ACADM_HUMAN | Medium-chain specific acyl-CoA dehydrogenase, mitochondrial |
| 2 | P55263 | ADK_HUMAN | Adenosine kinase |
| 3 | P51857 | AK1D1_HUMAN | 3-oxo-5-beta-steroid 4-dehydrogenase |
| 4 | P02647 | APOA1_HUMAN | Apolipoprotein A-I |
| 5 | P17405 | ASM_HUMAN | Sphingomyelin phosphodiesterase |
| 6 | Q9Y276 | BCS1_HUMAN | Mitochondrial chaperone BCS1 |
| 7 | P53004 | BIEA_HUMAN | Biliverdin reductase A |
| 8 | Q14790 | CASP8_HUMAN | Caspase-8 |
| 9 | Q9UPV0 | CE164_HUMAN | Centrosomal protein of 164 kDa |
| 10 | O15519 | CFLAR_HUMAN | CASP8 and FADD-like apoptosis regulator |
| 11 | Q96H96 | COQ2_HUMAN | 4-hydroxybenzoate polyprenyltransferase, mitochondrial |
| 12 | P08574 | CY1_HUMAN | Cytochrome c1, heme protein, mitochondrial |
| 13 | Q16854 | DGUOK_HUMAN | Deoxyguanosine kinase, mitochondrial |
| 14 | P54098 | DPOG1_HUMAN | DNA polymerase subunit gamma-1 |
| 15 | Q96RP9 | EFGM_HUMAN | Elongation factor G, mitochondrial |
| 16 | P07992 | ERCC1_HUMAN | DNA excision repair protein ERCC-1 |
| 17 | P16930 | FAAA_HUMAN | Fumarylacetoacetase |
| 18 | P07954 | FUMH_HUMAN | Fumarate hydratase, mitochondrial |
| 19 | P10912 | GHR_HUMAN | Growth hormone receptor |
| 20 | Q04446 | GLGB_HUMAN | 1,4-alpha-glucan-branching enzyme |
| 21 | P22830 | HEMH_HUMAN | Ferrochelatase, mitochondrial |
| 22 | Q9UG01 | IF172_HUMAN | Intraflagellar transport protein 172 homolog |
| 23 | P05231 | IL6_HUMAN | Interleukin-6 |
| 24 | Q96J02 | ITCH_HUMAN | E3 ubiquitin-protein ligase Itchy homolog |
| 25 | P78504 | JAG1_HUMAN | Protein jagged-1 |
| 26 | Q00266 | METK1_HUMAN | S-adenosylmethionine synthase isoform type-1 |
| 27 | P45985 | MP2K4_HUMAN | Dual specificity mitogen-activated protein kinase kinase 4 |
| 28 | P39210 | MPV17_HUMAN | Protein Mpv17 |
| 29 | O75648 | MTU1_HUMAN | Mitochondrial tRNA-specific 2-thiouridylase 1 |
| 30 | Q16236 | NF2L2_HUMAN | Nuclear factor erythroid 2-related factor 2 |
| 31 | P00480 | OTC_HUMAN | Ornithine carbamoyltransferase, mitochondrial |
| 32 | Q7RTS3 | PTF1A_HUMAN | Pancreas transcription factor 1 subunit alpha |
| 33 | Q9P2J5 | SYLC_HUMAN | Leucine--tRNA ligase, cytoplasmic |
| 34 | P56192 | SYMC_HUMAN | Methionine--tRNA ligase, cytoplasmic |
| 35 | P35625 | TIMP3_HUMAN | Metalloproteinase inhibitor 3 |
| 36 | P01375 | TNFA_HUMAN | Tumor necrosis factor alpha |
| 37 | P25445 | TNR6_HUMAN | Tumor necrosis factor receptor superfamily member 6 |
| 38 | O14763 | TR10B_HUMAN | Tumor necrosis factor receptor superfamily member 10B |
| 39 | Q8IWV7 | UBR1_HUMAN | E3 ubiquitin-protein ligase UBR1 |
| 40 | P09327 | VILI_HUMAN | Villin-1 |
